# In situ observation of nanolite growth in volcanic melt: A driving force for explosive eruptions

**DOI:** 10.1126/sciadv.abb0413

**Published:** 2020-09-23

**Authors:** Danilo Di Genova, Richard A. Brooker, Heidy M. Mader, James W. E. Drewitt, Alessandro Longo, Joachim Deubener, Daniel R. Neuville, Sara Fanara, Olga Shebanova, Simone Anzellini, Fabio Arzilli, Emily C. Bamber, Louis Hennet, Giuseppe La Spina, Nobuyoshi Miyajima

**Affiliations:** 1 Institute of Non-Metallic Materials, Clausthal University of Technology, Zehntner Str. 2a, 38678 Clausthal-Zellerfeld, Germany.; 2 School of Earth Sciences, University of Bristol, Queens Rd, Bristol BS8 1RJ, UK.; 3 Bayerisches Geoinstitut, Universität Bayreuth, 95440 Bayreuth, Germany.; 4 ESRF - The European Synchrotron, 71 avenue des Martyrs, CS 40220, 38043 Grenoble Cedex 9, France.; 5 ISMN-CNR, Istituto per lo Studio dei Materiali Nanostrutturati UOS di Palermo, via Ugo La Malfa, 153 90146 Palermo, Italy.; 6 IPGP-CNRS, 1 rue Jussieu, 75005 Paris, France.; 7 Institute of Mineralogy, University of Göttingen, Goldschmidtstr. 1, 37077 Göttingen, Germany.; 8 Diamond Light Source Ltd., Harwell Science and Innovation Campus, Didcot OX11 0DE, UK.; 9 Department of Earth and Environmental Sciences, University of Manchester, Oxford Rd, Manchester M13 9PL, UK.; 10 CEMHTI-CNRS, 1D Avenue de la Recherche Scientifique, 45071 Orléans Cedex 2, France.

## Abstract

Although gas exsolution is a major driving force behind explosive volcanic eruptions, viscosity is critical in controlling the escape of bubbles and switching between explosive and effusive behavior. Temperature and composition control melt viscosity, but crystallization above a critical volume (>30 volume %) can lock up the magma, triggering an explosion. Here, we present an alternative to this well-established paradigm by showing how an unexpectedly small volume of nano-sized crystals can cause a disproportionate increase in magma viscosity. Our in situ observations on a basaltic melt, rheological measurements in an analog system, and modeling demonstrate how just a few volume % of nanolites results in a marked increase in viscosity above the critical value needed for explosive fragmentation, even for a low-viscosity melt. Images of nanolites from low-viscosity explosive eruptions and an experimentally produced basaltic pumice show syn-eruptive growth, possibly nucleating a high bubble number density.

## Teaser

This study shows how a few nanometer-sized crystals markedly increase the viscosity of a magma leading to explosive eruptions.

## INTRODUCTION

From the devastation of Mt. St. Helens in 1980 (*1*) to the near catastrophe of Pinatubo in 1990 (*2*) and the relatively small eruption of the Icelandic volcano Eyjafjallajökull in 2010 (*3*, *4*), it is clear how easily our modern way of life can be severely disrupted by explosive, volcanic activity (*5*). Yet, this pales into insignificance compared to the devastation that would be caused by an eruption of Vesuvius similar to 79 CE (*6*) in such a highly populated area or a Tambora 1815 style event, which led to an average drop in global temperature of 1.5° to 2°C and “the year without a summer” in 1816 (*7*). Statistically, there is a 10 to 50% chance of a violent eruption bigger than Tambora during the 21st century, possibly erupting twice the volume of ash (*8*).

The most violent volcanic eruptions on Earth are commonly referred to as Plinian and release an enormous amount of energy in a sustained explosive event that can eject ash and gas into the stratosphere and over several kilometers on time scales of minutes to hours or days. Such sudden energy release is caused by the subtle interplay between an exsolving volatile phase, the mechanism of gas escape, but bulk magma viscosity plays a critical role in this process (*9*).

Viscosity describes a fluid’s internal resistance to flow. A magma consists of a liquid silicate melt containing dissolved gas (mainly H_2_O, CO_2_, and S) and variable amounts of crystals. If the magma is relatively low viscosity (very fluid), then most of the exsolving buoyant volatile bubbles have a good chance of escaping before that magma nears the surface, averting an explosive outcome. At low crystal content, the bulk magma viscosity is dominated by the melt chemistry, and the small crystal contribution can be ignored. In this case, the viscosity of a silicate melt can vary by orders of magnitude depending on temperature, chemical composition, and water content (*10*). Melt viscosity generally increases for a natural ascending magma as the temperature cools and as water is exsolved from the melt due to decompression. The water loss also markedly increases the liquidus temperature and, as more crystals grow, they eventually “lock up” the system and magma flow in the conduit becomes non-Newtonian. At the same time, there is less melt to host all of the volatiles and it fractionates to a higher silica content further increasing the viscosity. The exsolution of gas increases the buoyancy of magma by reducing its density, leading to an upward acceleration, which, in turn, induces further decompression degassing in a runaway feedback mechanism. In volatile-rich, silica-rich dacitic to rhyolitic magmas, this combination of circumstances commonly leads to volcanic explosions characterized by magma fragmentation and ash (*11*–*13*). The conditions for explosive fragmentation depend on a few factors such as the time scale of magma deformation and the relaxation time of the melt, but it is generally considered to require a minimum effective bulk viscosity of 10^6^ to 10^7^ Pa s (*11*–*13*), which is easily attained for silicic compositions.

Basaltic volcanoes generally provide a stark contrast to their high-silica cousins, showing relatively low explosivity even if they have a high volatile content, and they tend to form lava lakes and flows with occasional Strombolian fire fountaining. This gentle, effusive, or low explosive behavior is commonly attributed to their low viscosity, reflecting their low-silica composition, high eruption temperature, and lower crystal content. However, a clear paradox has been identified where low-viscosity magmas have been implicated in several major explosive eruptions (*14*–*19*). Particularly enigmatic are the geologically recent Plinian and sub-Plinian eruptions of “low-viscosity” magmas such as the Masaya volcanic system, Nicaragua (*20*); Tarawera 1886, New Zealand (*17*); Sunset Crater, USA (*21*); Tofua, Tonga (*22*); Pantelleria and Phlegrean Fields (*23*, *24*); and Tambora 1815, Indonesia (*25*). Perhaps, more worrying are unexplained changes in eruptive style for basaltic volcanoes such as Mt. Etna (Italy), including the destructive Plinian eruption of 122 BCE (*14*, *15*, *17*, *19*). One common explanation for this explosivity is late stage, rapid growth of small crystals in the melt (typically, 1 to >100 μm) referred to as microlites. At a particular volume percent (vol. %), which is strongly dependent on their aspect ratio (*26*), crystals can lock up the system, causing a rapid increase in the bulk magma viscosity (*26*) and, under typical strain rate conditions (*11*), can reach the fragmentation threshold, possibly even for the lowest viscosity melts such as basalt. However, for many Plinian and sub-Plinian eruptions, the role of crystals (microlites or bigger) is controversial since their volume percent appears to be too low, e.g., Tambora (*25*). Even for alkaline and peralkaline volcanoes such as Mt. Etna, Vesuvius, Phlegrean Fields and Pantelleria (Italy), Ethiopian plateau, and Kenya rift, this mechanism still requires some special pleading (*27*).

At a different size scale, crystalline nanoparticles (usually referred to as nanolites) are increasingly being discovered in geological samples over a wide range of environments of Earth’s surface (*28*) and in quenched glasses from erupted volcanic rocks (*29*–*31*) as well as experimental run products (*32*, *33*) using transmission electron microscopy (TEM) and Raman spectroscopy. Mujin *et al*. (*31*) have recently documented nanolites with diameters less than ~30 nm, which they refer to as “ultrananolites” that coexist with “small” often euhedral “microlites” (these can still be <1 μm). However, there is an important discontinuity in the size distribution (*31*), suggesting that they are not simply part of a continuum of crystal growth and that the microlites are possibly the product of Ostwald ripening (*34*) rather than a response to further undercooling. Outside of Earth sciences, nanoparticles have been of growing interest for the past 20 years, particularly in the fields of material and biomedical sciences. In relation to this current study, it has become well established that nanoparticles can have an effect on fluid rheology, which is hugely disproportionate to their small size and volume percent in a melt or suspension (*35*, *36*).

Hence, this raises the questions: Can nanolites play a substantial role in magma viscosity, promoting the fragmentation of magmas during cooling and ascent? In particular, can they have a previously unanticipated role in controlling the “eruption style” of low-viscosity magmas? Thermomechanical and differential scanning calorimetry analyses on geological samples and observations of experimental products have already hinted at the possibility that these particles can increase the magma viscosity and potentially interact with bubbles (*30*, *32*, *37*–*40*). There is clearly a need to understand the mechanisms that govern the formation of nanolites in geological systems, their relationship to larger microlites and bubbles, and their systematic effect on magma rheology.

Fast magma ascent and high undercooling (∆*T*) is crucial to produce rapid syn-eruptive crystallization within the conduit where fragmentation occurs (*41*). Therefore, in situ investigation of nanolite formation at realistic time scales (the first few hundreds of seconds of cooling and with a time scale resolution on the order of milliseconds) would allow structural observation of the incipient dynamics of nucleation. This may be before or coincident with the rapid microlite growth in basaltic magmas that has been observed using four-dimensional (4D) synchrotron x-ray microtomography (*41*). With this in mind, we have focused on the same Mt. Etna basalt as used by Arzilli *et al*. (*41*) to perform our in situ investigation on nanolite formation.

To address the question “Can the crystallization of nanolites explain the switch from effusive to explosive behavior, particularly in low-viscosity magmas?,” we designed a multifaceted approach: (i) determine whether nanolites are indeed present in various explosive low-viscosity eruptions; (ii) in situ experimental observations to determine whether nanolites can be generated during magma ascent; (iii) analog experiments to quantify the effect of nanolites on the bulk viscosity of a suspension analogous to a magmatic system; and (iv) degassing experiments to study the relationship between nanolite formation and bubble nucleation mechanism as a tracer of nanolite involvement. Our analysis also develops ideas relating to the effect of agglomeration of nanoparticles in suspensions. Techniques are detailed in Method and Materials and include the following: identification of nanolites in natural rocks using scanning TEM (STEM) and Raman spectroscopy; the in situ investigation of nanolite growth during undercooling used synchrotron-based x-ray diffraction (XRD), small-angle x-ray scattering technique (SAXS), and wide-angle x-ray scattering (WAXS); analog experiments using standard rotational rheometry to measure the viscosity of a suspension of silicon oil (a low-viscosity Newtonian fluid) and SiO_2_ nanoparticles; and simultaneous differential scanning calorimetry-thermogravimetric analyzer (DSC-TGA).

## EXPERIMENTAL RESULTS

### Identification of nanolites

**Fig. 1:**
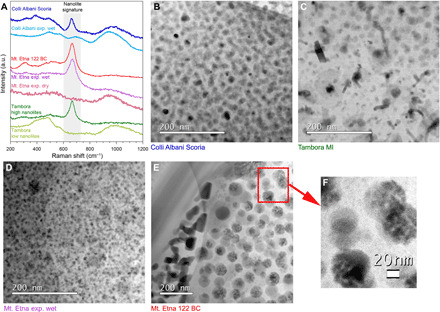
Nanolites in natural and experimental samples. The Raman spectra in (**A**) represent ~1000-nm areas of the nanolite bearing glass seen in the STEM images (**B** to **E**), as well other nanolite-free areas or samples that have been experimentally melted and quenched. The broad silicate bands at ~500 and ~1000 cm^−1^ are characteristic of nanolite-free glass, while the sharp peak at ~670 cm^−1^ has been attributed to FeO-bearing nanolites (*33*). Samples in (B) phonotephrite from Colli Albani, (C) trachy-andesite from Tambora, and (D) a hydrous experimental Mt. Etna basalt sample all contain 4 to 5 volume % of nanolites around 10 to 20 nm in size. In (E), a natural sample collected from the 122 BCE eruption of Mt. Etna shows two sets of nanolites; one solid (on the left adjacent to a plagioclase crystal) while the others appear to be agglomerates, enlarged in (**F**). The latter are more typical for the 122 BCE eruption. These agglomerates represent 13 to 20 volume % of the imaged samples, although it should be noted that the individual finer 5-nm aggregated particles in (F) (corrected for the intergranular melt) would represent <5 volume %. STEM wafers are ~100-nm thick. a.u., arbitrary units.

In Fig. 1, we present STEM images documenting previously unidentified examples of 20- to 50-nm nanolites in the glass from two low-viscosity Plinian eruptions of Mt. Etna 122 BCE (Italy) (*15*), Tambora 1815 (Indonesia) (*25*), and a sub-Plinian event that occurred at Colli Albani volcano located in the ultrapotassic Roman Province (Italy) (*42*). Also represented are experimentally synthesized samples all of which are described in the Supplementary Materials. Raman spectra for the nanolite-bearing samples show the established “nanolite” peak (*33*) at ~670 cm^−1^ between the wider bands centered at ~500 and ~950 cm^−1^, which arise from vibrations related to the amorphous structure of the silicate glass. As these nano-sized particles are clearly crystalline (see Materials and Methods), they can correctly be referred to as nanolites [or the ultrananolites of (*31*)]. Also included in Fig. 1 are some corresponding Raman spectra for natural and experimental glasses that appear to be free of nanolites, at least at the resolution of the STEM. The factors controlling the sharpness and height of the nanolite signature are complex and discussed in the Supplementary Materials.

### Initial identification of nanoscale structure

Preliminary runs in the wire furnace and other published data (*33*, *43*) suggest that an undercooling ranging between 50° and 200°C could be the decisive factor triggering the formation of iron-bearing nanolites. These observations directed us to target a few critical conditions for the in situ x-ray studies.

**Fig. 2:**
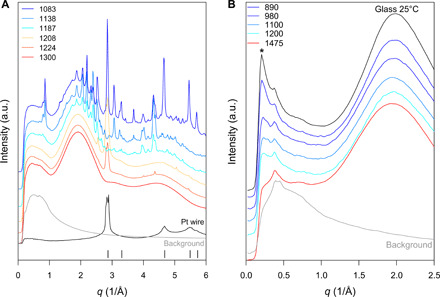
In situ synchrotron XRD patterns of molten Mt. Etna basalt during slow and fast cooling. In (**A**), the Mt. Etna basalt was cooled at 1°C min^−1^ in air starting from pure melt at 1300°C down to 1083°C. Results show the microlite crystallization for 60-s acquisitions at the temperatures indicated. The sharp Bragg peaks at 1224° and 1208°C represent a “spinel-structured” phase. Plagioclase peaks appear from 1198°C. The sharp peak observed at 2.9 Å^−1^ in the melt at 1224°C is the 2 2 0 Bragg diffraction peak of crystalline Pt (black tick positions), arising from incident x-ray beam impinging on the Pt-Rh10% heating wire as it contracts. The spectra in (**B**) are for a much faster cooling of Mt. Etna basalt from 1475°C to room temperature (in black) and then to each target temperature in the legend, in a pure Ar atmosphere. The asterisk (*) indicates the small-angle “nanolite peak,” which increases systematically with deeper undercooling. Note the absence of Bragg peaks. All data are displaced vertically. The background curve shows the XRD measurement performed with an empty cell and results from the Kapton window enclosing the wire furnace.

The initial in situ analytical technique used to test our question involved high-energy XRD (Fig. 2), collected on beamline I15 at Diamond Light Source (DLS; see Materials and Methods). Figure S1 illustrates the in situ XRD scattering intensity for the pure Mt. Etna basalt melt in air at 1300°C [above the liquidus estimated with rhyolite-MELTS (*44*), which is 1260°C at NNO + 2] and the glass when quenched to 25° from 1300°C at the maximum possible quench rate of ~10° to 20°C s^−1^, achieved by cutting power to the furnace. Notably, this glass provided the first sign of the small-angle peak (see fig. S1 and also Fig. 2B), which was not present in the melt. Determining the structure factors at larger angles combined with this sharp very-small angle scattering peak at ~0.3° in fig. S1 is consistent with the coexistence of a melt or glass matrix and a “separated phase” (see Materials and Methods) on the order of nanometers in size (*45*). The experiment proved that nanoscale phase separation was not present in the melt at 1300°C but formed with a rapid increase of undercooling. This led to a series of experiments at different cooling rates and, thereby, melt undercooling.

### Slow cooling rate and microlite crystallization

Figure 2A illustrates the in situ XRD intensities collected at different temperatures during slow, 1°C min^−1^ (0.017°C s^−1^) cooling of the Mt. Etna basalt from 1300°C under oxidizing conditions (i.e., in air). The appearance of clear diffraction peaks in the XRD pattern (e.g., 4.4, 5.1, and 5.4 Å^−1^) shows the onset of crystallization of the melt between ~1224° and ~1208°C. A structural refinement of these peaks indicates that the unit cell of first precipitating phases is probably magnetite. The extent of crystallization increases substantially at ~1187°C, where many sharper diffraction peaks are observed, which can be ascribed to the expected mineral crystallization sequence as predicted by rhyolite-MELTS (*44*) and previous experiments (*46*) (i.e., plagioclase then pyroxene with magnetite appearing dependant on the ƒO_2_). During this slow quench (over 1 to 4 hours), we observed no evidence for a peak at 0.3° (0.2 Å^−1^) that would imply nanoparticle formation. This is significant as it suggests that our observations (Fig. 2A) do not simply record the progression from small, nano-sized crystal nuclei to a crystal size of >100 nm.

### Fast cooling rates and nanolite formation

As nanoparticles are not formed at slow cooling rates of 0.017°C s^−1^ (Fig. 2A) but are observed at the faster (~10° to 20°C s^−1^) quench rate after a drop to 25°C (fig. S1), we explored the effect of faster cooling rates at temperatures more representative of those in a volcanic conduit. For some experiments, we also imposed a more reduced oxygen fugacity by using a pure Ar atmosphere. This ƒO_2_ is closer to natural magmatic conditions where silicate melt is stored at depth or during transport to Earth’s surface under conditions that are more reduced.

Figure 2B shows typical XRD spectra taken at the end of a series of relatively fast (~10° to 20°C s^−1^) single cooling steps from 1475°C down to four different target temperatures above and below the 1180°C liquidus, at 1200°, 1100°, 980°, and 890°C. Also shown for comparison is the pattern for the glass, after rapidly quenching from 1475° to 25°C under the same *f*O_2_ conditions. The diffuse XRD signal measured at 1475°C (red curve in Fig. 2B) is the characteristic of a nanolite-free melt. During the fast (single step) cooling to 1200°C, the small peak appearing at 0.2 Å^−1^ (* in Fig. 2B) indicates nanoparticle formation at slightly above the expected liquidus. As the target temperature for the single step is reduced to 1100°, 980°, and 890°C, the height of the small-angle peak at 0.2 Å^−1^ undergoes a systematic and substantial increase (* in Fig. 2B) revealing that nanophase separation is facilitated by larger undercooling (supersaturation) in the melt. The intensity of the small-angle peak in the 890°C undercooling measurement is very similar to that obtained for the glass rapidly quenched to room temperature. This also confirms that nanophase separation is clearly occurring above the onset of the glass transition temperature of the basaltic melt, which has been determined to be ~640°C for a slower cooling rate of 20°C min^−1^ (*47*). Although the data in Fig. 2B are focused on the smaller angle region, it is still clear that the Bragg peaks characteristic of Fig. 2A are absent, implying that the microlites do not have time to nucleate and grow in the few seconds (<30 s) covering cooling and acquisition.

### SAXS measurements

**Fig. 3:**
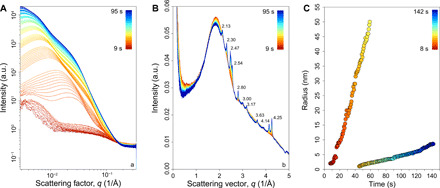
Nanolite growth with time. The spectra collected simultaneously at the (**A**) SAXS and (**B**) WAXS detectors show nanolite crystallization of Mt. Etna basalt experiment at a constant temperature of 950°C after fast cooling from 1600°C. The SAXS-WAXS scattering curves were acquired at a sampling interval of 0.5 s from 9 to 95 s of dwell time at 950°C. The color changes from hot (red) to cold (blue) with time. (A) The development of a SAXS signal with time. The lower red SAXS spectra were collected in the first 15 s. (B) WAXS scattering curves show initially no diffraction peaks (hot colors). Afterward, two peaks appear at 2.485 and 4.255 Å^−1^ after 15-s dwell time. The peak intensity increases slowly between 15 and 23 s. Last, the pattern shows a fast increase of multiple peaks at 2.128, 2.316, 2.47, 2.48, 2.51, 2.554, 2.806, 3.004, 3.173, 3.634, 4.135, and 4.144 Å^−1^. In general, peak intensity increases with time, whereas the peak width decreases. Numbers show the position of the peak. In (**C**), the evolution of the nanolite radius with time for the two populations of nanolites is derived by modeling the SAXS patterns collected during nanolite crystallization (see the Supplementary Materials for details).

To gain better access to the small-angle peak region, hidden by the beamstop in Fig. 2 and fig. S1, SAXS-WAXS measurement was made on beamline I22 at DLS (UK). In fast acquisition mode, these techniques provide real-time analyses, on a time scale of seconds, rapidly probing the transient evolution of these small particles during cooling and crystallization. We show one example where we have determined the size and time scale of nanoparticle formation and growth in a sample rapidly cooled in a single step from well above the liquidus temperature (1600°C) and then held at 950°C while continuously collecting in situ SAXS-WAXS measurements at an extremely fast acquisition rate (every 0.5 s). The initial SAXS-WAXS patterns collected at 1600°C (Fig. 3, A and B) illustrates that the sample was fully molten and free of nanoparticles as demonstrated by the absence of both a SAXS signal and Bragg peaks in the WAXS region (Fig. 3B). During the first 15 s of in situ isothermal measurements at 950°C, there is the clear development of a symmetric band centered at 0.064 Å^−1^ in the SAXS region (Fig. 3A), while the WAXS spectra (Fig. 3B) are yet to show any clearly resolved Bragg peaks that would indicate sizable, well-formed crystals.

This local increase in Fe─O bonds might explain the low broad peak observed in some Raman spectra (Fig. 1). Modeling of the SAXS pattern (Fig. 3C) suggested that, during the first 20 s of experiment, the melt evolved into a suspension carrying spherical entities ranging between 2- and 15-nm radius. In addition, SAXS patterns collected within the first 10 s are characterized by a rapid increase in intensity at low *q* (*q* < 0.22 nm^−1^, Fig. 3A). SAXS patterns collected during further 10 s show prominent local maximum developed at *q* ~ 0.25 nm^−1^ with continued increase in the intensity at lower *q* < 0.12 nm^−1^. The rapid increase in the intensity at lower *q* likely originates from the formation of particle aggregates in the melt described by a dense-packed mass fractal dimension (*48*). The subsequent SAXS profiles demonstrate continuous growth of particles up to 50 nm before size information is lost as it increases outside the experimental *q* minimum range (Fig. 3, A and C). After 30 s into the isothermal crystallization, the second population of spherical polydisperse particles emerges in the melt. They measure ~8 nm by the end of the cycle. Simultaneously, we observed (Fig. 3B) the appearance of broad diffraction peaks in the WAXS pattern at 2.485 and 4.255 Å^−1^ appearing after 15 s of holding the temperature at 950°C. (Fig. 3B). This is also consistent with the lack of Bragg peaks in the first 10 s of cooling in Fig. 2B and the agglomeration seen in the natural Mt. Etna sample in Fig. 1 (E and F). The emerging peaks are identified as magnetite and hematite Bragg reflections (the peak at 3.173 A^−1^ could not be assigned unambiguously). The emergence of hematite crystallites is the probable source of the second population of particles seen in the SAXS profiles and may represent oxidation at the sample-air interface. Overall, the peak intensity increases with time, whereas the peak width decreases suggesting an increase in the degree of crystallinity in the melt. The modeling of the SAXS pattern together with the observation of crystallinity from the WAXS signal suggested that we were observing the growth of magnetite nanolites with a maximum size of 50 nm, achieved after ~60 s of dwell time at 950°C (Fig. 3). This is consistent with the images for natural samples, including Mt. Etna in Fig. 1E.

### Viscosity of magma analogs with nanoparticles

**Fig. 4:**
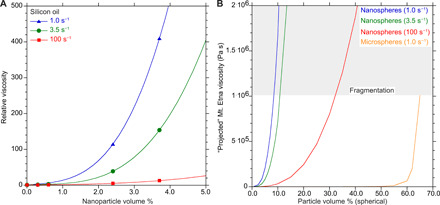
The nanolite effect on viscosity. (**A**) Illustrates the measured relative viscosity (η*_r_* = η_suspension_/η_oil_) of analog magma made of silicon oil and spherical SiO_2_ nanoparticles of ~15-nm diameter at different shear rates. In (**B**), the relative viscosity from (A) is used to calculate the expected viscosity of nanolite-bearing melt with an initial viscosity of 200 Pa s (η_projected_ = η*_r_* × 200 Pa s), equivalent to Mt. Etna basalt at preeruptive conditions (temperature and water content). We also show the equivalent curve for microlites, using the relative viscosity of Mader *et al*. (*26*) for SiO_2_ microspheres, also in silicon oil at a shear rate of 1 s^−1^. It is clear that <10 volume % of nanoparticles is equivalent to >60 volume % of microparticles and can raise the viscosity to ~10^6^ Pa s required for magma fragmentation (*41*). The traditionally calculated viscosity for <10 volume % microparticles according to the Krieger and Dougherty (*75*) model, as described by Mader *et al*. (*26*), would barely show above the *x* axis in these figures.

To quantify the potential physical effect of such nanolites in magmas, we performed viscosity measurements using 15-nm spherical silica nanoparticles dispersed in silicon oil as a magma analog. Four samples were prepared, characterized by a volume percent (ϕ) of nanoparticles of 0.3, 0.6, 2.4, and 3.7 volume %. This low volume % range is relevant because the amount of iron in the low-viscosity magmas such as Mt. Etna basalt and Colli Albani phonotephrite is ~10 weight % (wt %) (*15*, *42*) and, assuming the complete extraction of iron from the melt to form single crystal magnetite nanolites, the maximum volume percent that could be formed would be ~7 volume %, given the higher density of magnetite (5.2 g cm^−3^) than that of a basalt (~3.0 g cm^−3^). To explore the possibility of non-Newtonian behavior, the nanolite suspensions were measured at steady shear rates in the volcanologically relevant range of 1 to 100 s^−1^. As illustrated in Fig. 4A, the relative viscosity (η*_r_* = η_suspension_/η_oil_) of the suspension with the highest volume percent of nanoparticles (still only 3.7 volume %) was ~400×, ~150×, and 15× higher than the viscosity of the silicon oil measured at shear rates of 1.0, 3.5, and 100 s^−1^, respectively (Fig. 4A). As shown in Fig. 4B and on the basis of the detailed modeling calculations presented in the Supplementary Materials, for a 1.0 s^−1^ shear rate, ~65 volume % of microparticles (in orange) is required to reach the maximum packing density (ϕ*_m_*) and lock up the suspension. The same effect on viscosity is produced by just ~8 volume % of nanoparticles. Even at the lowest volume percent of particles (0.3 volume %), the viscosity of our nanosuspensions was still found to display non-Newtonian behavior as the viscosity decreased with increasing shear rate $\dot{\gamma }$ from 1 to 100 s^−1^, whereas a microsuspension with 0.3 volume % would behave as a Newtonian fluid (*26*). In Fig. 4B, the relative viscosities reported in Fig. 4A have been translated to absolute viscosities of a nanolite-bearing basalt by calculating the viscosity of the liquid phase on a chemical basis (*49*) at eruptive conditions [<1070°C (*41*, *46*)] and considering the physical effect of nanolites measured at different shear rates (Fig. 4A). The result of the modeling suggests that the magma approaches the fragmentation threshold (*41*) of ~10^6^ Pa s at 8, 11, and 32 volume % of particles at shear rates of 1.0, 3.5, and 100 s^−1^, respectively.

### Degassing and pumice formation experiments

Although we have demonstrated that nanolites can form in response to undercooling by a simple drop in temperature, in the case of an ascending magma, undercooling is more likely to be related to degassing and the resultant increase in the liquidus temperature. Related to this, it has been suggested (*30*) (and references therein) that nanolites play a role in the heterogeneous nucleation of bubbles with important implications for the dynamics of volcanic eruptions and their style. In particular, the formation of nanolites can induce the nucleation of bubbles and cause them to remain coupled to the rising magma. Such distinctive vesicle patterns could be used to fingerprint the influence of syn-eruptive nanolite formation (*50*), even if the nanolites are transient and subsequently reabsorbed or “ripened” to give microlites. The in situ XRD techniques used in this study are difficult to adapt to a pressurized system that would allow controlled undercooling by degassing. We therefore explored this mechanism by using a hydrous version of the same Mt. Etna melt and combining a DSC-TGA with TEM and Raman spectroscopy analyses.

**Fig. 5:**
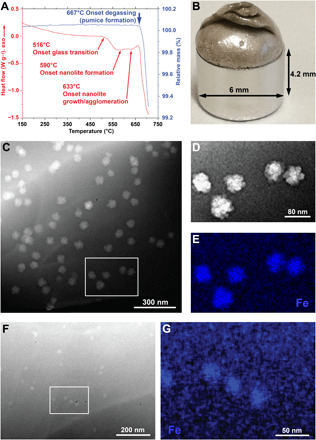
Nanolite and pumice formation. (**A**) Simultaneous thermal analysis (STA) showing the heat flow (red) and weight loss (blue) during heating of a nanolite- and bubble-free Mt. Etna hydrous (H_2_O = 1.48 wt %) glass (experiment 1). The different thermal events are reported together with the onset of degassing that led to pumice formation in (B). (**B**) Picture of the recovered sample after STA measurement in (A). Note the considerable increase in the sample volume due to the development of a very high porosity. Before the measurement, the sample was a doubly polished dense nanolite- and bubble-free glass measuring 3 mm by 3 mm by 2 mm. Photo credit: Danilo Di Genova. (**C**) High-angle annular dark-field (HAADF)–STEM image of the pumice in (B). The rectangle shows the investigated area in (**D**) and (**E**) with STEM-EDS that suggests nanolites are Fe rich (E). (**F**) HAADF-STEM and (**G**) STEM-EDS images of the STA experiment 2 (stopped at 620°C). Note that the number density of nanolites is lower and their size is smaller than in (**C**).

In experiment 1, we subjected a hydrous glass [H_2_O = 1.48 wt % (*47*)] to a constant heating of 30 K min^−1^ in a controlled atmosphere (N_2_) to avoid the oxidation of the iron dissolved in the melt. During heating, we observed (Fig. 5A) the onset of the glass transition region at 516°C, which indicated the beginning of the transition from glassy to liquid state of our sample. Subsequently, at 590°C, we observed a weak exothermic event that we associated with the incipient formation of nanolites (see experiment 2). Following this exothermic event, a more important new exothermic event appeared at 633°C, which we associate with the growth and possibly agglomeration of nanolites. This event was followed by a vigorous endothermic event and a rapid loss of weight from the sample. The endothermic event marked the melt degassing and basaltic pumice formation (Fig. 5, A and B, and fig. S8, A and B).

The collected Raman spectrum (fig. S8C) is compatible with a glass matrix characterized by the presence of FeO-bearing nanolites. The pumice was subjected to a high-angle annular dark-field (HAADF)–STEM analysis (Fig. 5, C and D), and the results confirmed the presence of agglomerates of FeO-bearing nanolites across the sample having a shape and size (*r* ~ 40 nm) similar to those of the products erupted during the 122 BCE Plinian eruption of Mt. Etna (Fig. 1E). To the best of our knowledge, this is the first experimental evidence of the formation of a nanolite-bearing basaltic pumice.

In experiment 2, we carried out a second DSC-TGA experiment where the heating of the hydrous sample was stopped at 620°C, which corresponds to a temperature higher than that of the incipient formation of nanolites (590°C in Fig. 5A) but lower than that of their growth/agglomeration. Results showed (Fig. 5, F and G) the presence of more dispersed FeO-bearing nanolites across the sample with a size (*r* ~ 15 nm) lower than observed in experiment 1. The sample recovered at the end of the experiment was visually unaffected with respect to its initial state (i.e., a dense black glass). The Raman spectrum (fig. S9C) for this black glass is substantially different from that of the pumice, and the low nanolite content reduces the 670 cm^−1^ peak to resemble a typical nanolite-free basaltic glass (*30*).

## DISCUSSION

The established crystallization sequence for the Mt. Etna natural basalt has either plagioclase or clinopyroxene on the liquidus around 1120° to 1075°C depending on the pressure/water content, with an iron oxide (titanomagnetite) appearing below 1020°C (*46*). At the unrealistically high oxidizing condition of experiments run in air, the initial phase is an iron oxide and the liquidus for this mineral can rise to above 1240°C (*51*) [rhyolite-MELTS (*44*) suggesting 1312°C at the Hematite-Magnetite buffer]. This is related to the high ratio of Fe^3+^/Fe^2+^ in the melt, although the phase is still a (titano) magnetite (*51*) rather than the more oxidized iron oxide phase hematite. In our study, we are unable to analyze the chemical composition of the nanoparticles, but the Bragg patterns are consistent with a range of iron oxides. Consequently, we will use the term magnetite given the quality of the Bragg patterns. In some cases, the peaks are more specifically consistent with titanomagnetite.

There is no sign of a nanolite peak in the first XRD pattern measured for pure melt at 1300°C after 1 hour or in subsequent patterns during slow cooling and crystallization. The 1300°C measurement was identical to the diffuse liquid measurement at 1600°C. However, the XRD appears to show the growth of “magnetite” between 1224 and 1208°C within 1 to 2 hours, indicating that the liquidus is below 1300°C. This suggests that (i) the crystals are all microlites and that nanolites did not form at the slower cooling rate, (ii) nanolites formed and were reabsorbed within 1 hour, or (iii) they have developed into microlite sized features by reorganization or Ostwald ripening, which is particularly efficient below 10 μm (*52*). When the 1300°C pure crystal free melt is quenched by turning off the furnace, the cooling is three orders of magnitude faster (between 10° and 20°C s^−1^) and a nanolite peak is found in the subsequent glass. Given a magnetite liquidus between 1300° and 1250°C and a glass transition temperature of ~700°C (*47*), these nanolites had less than 40 s to develop.

The next experiments (in Fig. 2B) were cooled at a fast rate similar to a “quench” (~10° to 20°C s^−1^) but were stopped at some specific temperatures to collect a pattern and were performed in a controlled argon atmosphere more appropriate to natural conditions (NNO + 1). This ƒO_2_ will lower the liquidus to between 1260° and 1140°C [(*53*) and rhyolite-MELTS (*44*)], and plagioclase and/or clinopyroxene will form at around the same time or possibly before the magnetite at NNO. These experiments demonstrate the growth of a nanolite phase as a precursor to microlites in the first 2 to 30 s of undercooling (∆*T* 40° to 250°C) below the liquidus. This clearly demonstrates that nanolite formation could be an important process in the conduit during a Plinian and sub-Plinian eruption, especially if a rapid increase of undercooling is induced through fast magma ascent reaching thermodynamic conditions favorable for the rapid nucleation of nanolites (∆*T* = 40° to 250°C). This range of undercooling is expected during fast magma ascent (5 to 50 m s^−1^) in the conduit, as constrained by both experiments and numerical simulations for a basaltic Plinian eruption at Mt. Etna (*41*).

For the sample held at 950°C in Fig. 3, the SAXS examination of the first ~150 s of undercooling by 160° to 190°C in air documents the continuous growth of nanolites to 50 nm before a second series of nanoscale features develop (Fig. 3). The contemporaneous WAXS spectra (Fig. 3B) confirm that these are crystalline magnetite at least after the initial growth stage, and the nanoscale is also confirmed by the broadness of the Bragg peaks, which sharpen with time as the crystals increase in size. However, as expected, a sample heated from room temperature up to 950°C (in N_2_) over 20 min displayed all of the equilibrium microlite phases and none of the nanolite features. Again, this shows the role of nanolite formation at the rapid time scales of magma ascent.

It should also be noted that our in situ experiments are water-free, and this has some important implications when applying these ideas to nature. First, our in situ experiments induced a rapid thermal perturbation over a time scale of a few seconds to experimentally simulate an “undercooling effect” (*∆T* between 40° and 250°C) and promote the sudden formation of nanolites at eruptive temperatures. However, for wet magmas in nature, undercooling is controlled primarily by the decompression rate, which, in turn, controls the exsolution of volatiles and thereby increases the liquidus temperature of the magma, even at a relatively constant temperature (*54*–*56*). Therefore, continuous decompression during magma ascent can favor continuous vesiculation and crystallization. This implies that continuous nucleation events of bubbles and nanolites can occur during magma ascent and that nanolite crystallization can promote further volatile exsolution, increasing the bubble number density as documented in the pumice formation experiment (Fig. 5).

Our experimental production of “dry” nanolites may also seem at odds with the conclusions of Di Genova *et al*. (*33*) who suggested that >3 wt % water created conditions favorable for nanolite formation in the wide compositional dataset they examined. The role of water is probably important as it increases the diffusivity, which will control any nucleation process. However, diffusivity is also controlled by temperature. Hence, given a range of samples with different compositions (including water), temperatures, and quench rates, it is likely that the Di Genova *et al*. (*33*) sample set has probed a different “diffusion window” to this study.

Our viscosity measurements demonstrate a phenomenon that is familiar in material science (*35*) but remains poorly understood, where the rheological behavior of the suspension markedly changes with the size of the solid particles. We measured the viscosity at volcanologically relevant shear rates, which are lower than those commonly used in the nanofluids literature. Our measurements show that the increase in viscosity becomes larger as the shear rate decreases (i.e., “shear thinning” behavior). Our data thus further expand the measurement range to relatively low shear rates (Fig. 4), where the measured increase in viscosity is maximum. Numerical modeling (*35*) suggests that the number density of particles *n* (*n* = *N*/*V* where *N* is the number of particles in a given volume *V*) plays a crucial role in affecting the fluid dynamics of the suspension. For the same volume % of particles (ϕ), the value of *n* is much higher for nanoparticles compared to microparticles. This results in a smaller distance between two neighboring particles that may affect the local flow of liquid between particles, the bulk viscosity of the suspension, and promotes the onset of non-Newtonian behavior at low volume percent of nanoparticles (i.e., ϕ < 5 volume %). This process can be neglected when the liquid phase carries a low volume % of microparticles since the average distance between particles is on the order of microns (*35*).

This agrees with results from other studies performed on suspensions and molecular dynamics simulations (*35*, *57*–*59*). These studies suggested that a moving and isolated nanoparticle in a fluid induces velocity fluctuations in the carrier medium through the occurrence of toroidal vortexes near the surface of the nanoparticles. These fluctuations have a characteristic diameter in the order of the nanoparticle dimension, and their occurrence requires the dissipation of the energy stored in the suspensions and this ultimately increases the viscosity of the suspension (*35*).

In addition to this, there is the effect of nanoparticle agglomeration, a phenomenon well studied in materials science (*36*) and possibly observed in the natural (Fig. 1) and experimental (Fig. 5) samples. Because of their large surface area, nanoparticles tend to agglomerate through van der Waals interactions (*36*). This agglomeration process will incorporate the carrying fluid on the surface of each nanoparticle into the “effective volume” of the solid, which, in turn, increases the viscosity of the nanosuspension. We modeled the evolution of the effective volume of the solid fraction as a function of the size of agglomerates, the volume of the liquid entrapped between particles, and the thickness of immobile liquid around each particle (see the Supplementary Materials). The simulation was carried out taking into consideration our observation that for ϕ ~ 4 volume % of nanoparticles and a shear rate of 3.5 s^−1^, the viscosity is increased by a factor of 10^2^ (Fig. 4). This would normally occur at ϕ ~ 60 volume %, if we swapped nano for any size of microparticles (see Fig. 4B). As a result, the effective volume percent of agglomerated particles would need to be 15× that of the particles themselves to have a similar effect on the viscosity. The model shows that a thickness of immobile liquid on the order of the particle radius leads to the required increase in the effective volume percent of solids. This also agrees with recent observations (*38*, *60*) of glasses containing nanocrystals. Agglomeration may also explain the shear-thinning effect, where the increase in viscosity is less at high shear rates. This may reflect the breaking up of the agglomerates as various attractive interactions (e.g., Van der Waals) are overcome (*61*). As there is a range of shear rates experienced within any volcanic conduit, the effect of nanolite agglomerates may be specific to certain zones.

If FeO-bearing oxide is the main nanolite phase producing this phenomenon in nature, then most low-viscosity magmas erupted on Earth such as basalt, phonotephrite, leucitite, phonolite, and pantellerite have a high enough FeO content to produce this low crystal volume percent (< 5 volume %), although other silicate phases would be required in more silicic magmas. Our results show that, when the magma undergoes nanolite crystallization (Fig. 3), its rheology would change markedly within seconds; this increase in viscosity and switch to non-Newtonian behavior (Fig. 4) would produce a dramatic effect on the eruptive behavior. Numerical modeling (*11*, *41*) suggests that these conditions are sufficient to exceed the maximum stresses that can be supported by the magma during the eruption, crossing the viscous-brittle regime and thereby inducing explosive eruptions.

Mujin *et al*. (*31*) have suggested that nanolites are syn-eruptive crystalline phases and argued that they crystallize when the undercooling of the magma increases rapidly near the surface due to degassing of volatiles. Barone *et al*. (*29*) showed Fe-enriched nanoparticles on the surface of ash particles and concluded that such nanoparticles may result from chemical reactions taking place at the bubble-melt interface in the shallow plumbing system. The authors suggested that this interface represented the proto-fragmentation surface of the future ash particles. This observation implies that brittle failure of magma is associated with nanolite formation during the eruption. Furthermore, recent in situ observations of bubble nucleation in silicate melts (*37*) and experimental results (*30*, *40*) show local mutual affinity between bubbles and iron oxides, resulting in the formation of bubble-oxides agglomerates. The recent observations from the literature and our experimental results suggest that nanolite occurrence could be a key trigger of explosive volcanic eruptions but might be difficult to detect in the eruptive products.

Multiple decompression events and subsequent undercooling of magma during a single eruption can induce repeated events of nanolite formation over time scales of a few seconds. We argue that nanolite crystallization is the incipient stage of microlite crystallization. Once these nanolites have crystallized, they may continue to form due to several undercooling events, alongside microlite crystallization. This explains the coexistence of microlites and nanolites. SAXS-WAXS results in Fig. 3 show that nanolites grew up to 50 nm in radius in ~60 s at 950°C (~1 nm s^−1^). We found that growth rates of iron oxide microlites in basalts at similar temperature (*62*) are at least one order of magnitude slower than that of nanolites. As such, rapid increase of undercooling is a key factor leading to the fragmentation of low-viscosity magmas. Other recent in situ observations of microlite crystallization in Mt. Etna basalt and numerical modeling of magma transport (*41*) revealed that the fast ascent of magma produces large undercooling between 80° and 175°C, which, in turn, drives unexpectedly rapid microlite formation on the time scale of minutes. This is possibly one way to increase the magma viscosity at Mt. Etna up to the fragmentation threshold of ~10^6^ Pa s. However, the precursor growth of nanolites on the time scale of seconds may be the trigger for the whole feedback cycle of rapid ascent induced by trapping gas in a melt that suddenly becomes very viscous. The DSC-TGA experiments (Fig. 5A) and the HAADF-STEM and STEM–energy dispersive X-ray spectroscopy (EDS) images (Fig. 5, C and D) demonstrate that we were able to capture the transition between a completely amorphous system (melt) and a system characterized by small and dispersed nanolites (Fig. 5, F and G), which then evolved into a nanolite- and bubble-bearing system (Fig. 5B and fig. S8, A and B) that resembles the product (i.e., pumice) of Plinian eruptions. If the nanolites survive in the matrix melt (as apparent in Fig. 1E), then lesser amounts of microlites would be required to lock up the Mt. Etna system, which could explain the eruption of Tambora with low microlite content (*25*) (see the Supplementary Materials for more details). However, an explosive eruption can still only occur if the magma deformation rate reaches the minimum conditions for its brittle fragmentation (*11*).

The results from our study may also explain observations in the literature of a substantial increase in the viscosity of terrestrial and extraterrestrial magmas at ϕ < 5 volume % recently observed (*32*, *38*, *63*) and of the dependence of maximum packing density of solid particles (ϕ*_m_*) on the particle size observed in magma analogs (*64*). As particle size is time dependent, we modeled the magma viscosity evolution as the crystal size evolves with time from nano- to microscale (see the Supplementary Materials). We found that at ϕ ~ 4 volume % of nanoparticles and a shear rate of 3.5 s^−1^, the viscosity is increased by a factor of 10^2^ and 10, respectively, 100 and 300 s after the nanolites appeared. The effect vanishes when the particles reach the microsize. Our isothermal, constant composition (nonfractionating) analog experiments using SiO_2_ nanoparticles and silicon oil clearly demonstrate the pure nanolite effect on viscosity. However, in natural molten systems where the crystallizing nanolite represents a phase separation, it is also clearly important to consider the additional chemical effect on matrix melt viscosity due to fractionation and the fall in temperature (*38*). This is especially important when Fe (or Ti) oxides are involved (*39*) and may also be exaggerated as local chemical gradients are established before reequilibration with the rest of the melt. To our knowledge, the only published studies using glassy samples to measure melt viscosity coupled with either Raman spectroscopy or TEM imaging are those of Liebske *et al*. (*32*), who actually found the presence of nanolites in their andesitic sample and suggested that they could be the reason for anomalous results, and a more recent study using Mt. Etna basalt and technical glasses (*38*). The evidence from these experimental studies suggests that the findings of our rheological measurements can also be applied to natural systems.

In this study, we have established that nanolite formation can contribute to a rapid and substantial increase in magma viscosity of several orders of magnitude during magma ascent. Under certain circumstances, this pronounced increase in magma rheology may trigger a sequence of events causing the magma to reach the fragmentation threshold of ~10^6^ Pa s and hence promoting an explosive eruption, even in low-viscosity magmas. It is also possible that nanolites are a ubiquitous feature of magmas but may have a transient effect as the nanolites induced by undercooling have time to grow to microlite size (~100 μm, fig. S9A). Where this occurs, the maximum packing fraction increases to that expected of microlite-bearing magmas (see fig. S9). It is possible that the aggregates are the transient precursors of microlites, which form by reorganization to align their crystal axes (*65*). This could explain the two nanocrystal types noted in Fig. 1E. Understanding the subtle factors that define the nanolite-controlled window between high (explosive) and low (effusive) viscosities may be fundamental in predicting the “unpredictable” switches in eruptive style at volcanoes such as Mt. Etna and major low-viscosity, low crystal eruptions such as Tambora.

## MATERIALS AND METHODS

### STEM imaging Fig. 1

The TEM foils (of approximate dimensions of 20 μm by 12 μm by 0.1 μm) were extracted using a combined SEM/focused ion beam instrument (FEI Helios Nanolab 600i, University of Bristol). A platinum deposit was made at each extraction site for protection before milling and thinning. The foils were subsequently loaded onto holey carbon films by ex situ liftout using an optical microscope and micromanipulator before analysis by TEM. Bright-field and HAADF-STEM images were collected at the David Cockayne Centre for Electron Microscopy (Material Sciences, Oxford University) using a Jeol ARF-200F operating at 200 kV with a cold field emission source and featuring 100-mm^2^ Centurion EDX detector and Gatan GIF Quantum 965 ER featuring dual electron energy-loss spectroscopy and energy-filtered transmission electron microscopy (EFTEM) capabilities.

### STEM-EDS imaging (Fig. 5)

The analysis of the experimental samples was carried out using scanning TEM (FEI Titan G2 80-200 S/TEM, Bayerisches Geoinstitut, University of Bayreuth) equipped with EDS consisting of four-channel silicon drift dectector (Bruker, QUANTAX EDS) and operated at 200 kV.

### Raman spectroscopy

Raman spectra were acquired using a Thermo Scientific DXR3xi Raman Imaging Microscope at the University of Bristol, School of Earth Sciences, using a 532-nm (green) doubled Nd:YVO4 DPSS excitation laser and a 900 lines mm^−1^ grating. Raman spectra were acquired between 200 and 1500 cm^−1^ with a 100× objective, 25-μm confocal pinhole, and a laser power of 3 mW. The Raman scattering was acquired for 5 s and averaged over 10 scans.

The nanolite peak (see Fig. 1A) represents the vibration of Fe─O bonds, but the position and sharpness are consistent with those bonds being in the FeO oxide mineral (possibly magnetite), suggesting that the nanoparticles are “crystalline” rather than features of the melt structure [e.g., (*66*)]. This crystallinity has also been confirmed by lattice images observed during HAADF imaging and by selected area electron diffraction during our STEM imaging. For some experimental hydrous Colli Albani samples that appear to have nanolites in the TEM image, the corresponding Raman spectrum characterized by a small broad feature slightly offset from 670 cm^−1^ rather than a clear sharp peak. This illustrates a point that the nanolites may have not developed any substantial macrocrystalline structure, even if the particles have formed sizeable nanoagglomerates. There will be several stages to their formation with initial meso-range order in the melt before nuclei form and enough molecules gather to be considered a crystal lattice.

### Strategies for in situ observations

#### *X-ray techniques*

Three different synchrotron-based x-ray spectroscopic techniques were used to document in situ nanolite formation in the Mt. Etna basaltic magma. XRD traditionally allows the identification of Bragg peaks from the angstrom scale (0.1 nm) planar repeat features in developing crystalline structures, but using a high-energy synchrotron source also offers a way to probe the disordered melt structure down to the scale of inter-atomic pairs. As well as atomic-scale ordering in the melt structure, this technique gave the first hint of a sharp, very-small angle scattering peak consistent with coexistence of nanometer-sized features in the melt matrix.

To further investigate the temporal in situ development of these small-scale features in more detail, a SAXS was used. SAXS is excellent for probing the characteristics of particles in the nanometer size range. However, it is a contrast method that measures the scattering signal derived from the difference between average electron density. Hence, while it can detect the phase boundaries that represent a density difference, it cannot directly distinguish between separation of crystals, immiscible melts, or even areas of amorphous medium range atomic order that might be present in an otherwise homogeneous melt (*67*) or clusters that represent pre-nuclei (*68*). However, it can be simultaneously combined with a WAXS, which provides a means to identify characteristic Bragg peaks associated with XRD from subnanometer scale crystalline lattice planes. Model fits of the SAXS spectra can return parameters that not only define the “particle size” but also their volume percent, number per unit volume, total surface area, and size distribution and whether the “particles” represent single entities or the agglomeration of smaller particles together, namely, how they interact with each other.

Our in situ observations were all made on melt taken above its liquidus temperature in a Pt-wire furnace (*69*) and then cooled under both oxidized and reduced oxygen fugacity (*f*O_2_) conditions. Both slow (1°C min^−1^) and fast (~10° to 20°C s^−1^) cooling rates were used to study the transient evolution of nucleation and crystallization at different degrees of undercooling below the magma liquidus temperature.

### Starting material for in situ experiments

The starting glassy material used for our XRD experiments is the composition reported and used in Polacci *et al*. (*70*) and obtained by melting volcanic products retrieved from the lower vents of the 2001 Mt. Etna eruption. The natural samples were crushed in a jaw crusher, powdered in a carbide ring mill, and melted in a thin-walled Pt crucible in a box furnace at 1400°C for 4 hours. Afterward, the melt was poured, in air, onto a steel plate and rapidly quenched to glass. This procedure was repeated twice to ensure sample homogeneity. For the experiments, the homogeneous glass was finely powdered using an agate sphere mortar grinder.

### Heating cell

High-temperature in situ measurements were made using a microheating cell (*69*) consisting of a 1-mm-diameter Pt-Ir 10% wire. A flattened region was formed at the midpoint of the wire, within which a ~1-mm bore was made to form the sample chamber. Each experiment has a new wire, which is calibrated so the temperature for a given power setting is known within ±5°C. For details on the temperature calibration procedure, see the study by Neuville *et al*. (*69*). To load the sample, a finely ground, powdered sample was loaded in the sample chamber and rapidly heated to 1600°C to obtain a pure, crystal-free melt. The sample was held at high temperature for a minimum of 30 min before running an experiment, to ensure equilibration at the correct oxygen fugacity and the removal of any crystalline features (nano or micro) that might have been present in the starting glass powder.

Movement of the wire due to heating is considerable. For slow cooling experiments, there is time to realign the synchrotron beam for each temperature. For fast cooling and acquisition, the position is precalibrated before the loaded sample comes into alignment at the temperature required for measurements. Only an approximate maximum cooling rate can be reported of ~16°C s^−1^. It depends on the starting point and range of temperatures and is unlikely to be strictly constant. However, the time taken for the wire hole to align and let the beam through confirms this cooling rate. Presaturation of the Pt-Ir wire with Fe-bearing melt (remove with HF) at appropriate conditions ensures minimal Fe-loss (or gain) during an experiment. For controlled-atmosphere experiments, the wire heater is enclosed by a chamber with Kapton windows to allow unimpeded access to the sample for the x-ray beam. Reduced conditions were achieved by passing pure Ar gas through the cell with a flow rate of ~1 liter min^−1^.

### In situ XRD measurements and total structure factors

Angle-dispersive XRD measurements were made for the high-temperature basalt melt at beamline I15 at the DLS, UK, at an energy of at 72.0 keV with an incident x-ray beam of wavelength λ = 0.1722 Å and collimated using a 70-μm W-pinhole. 2D XRD patterns of the high-temperature melts were measured using a PerkinElmer image plate detector. The in situ slow cooling rate measurements were performed by reducing the temperature by 5°C every 5 min. The XRD diffraction intensity, *I*(2θ), was collected as a function of scattering angle 2θ for 60 s at each time step. The in situ fast cooling rate measurements were performed using 1-s acquisitions. At the end of each run, the sample was remelted at 1600°C, and the pre- and postexperiment melt diffraction patterns were compared to ensure sample composition stability.

The data were calibrated using the diffraction pattern obtained for a LaB_6_ standard and reduced to 1D profiles accounting for geometrical effects and incident beam polarization using the Data Analysis WorkbeNch (DAWN) software suite. Structural refinement of the crystalline Bragg diffraction peaks was carried out using the Le Bail method in the program General Structure Analysis System (GSAS) (*71*). The liquid and glass total structure factors *S*(*q*) were obtained by normalization to the *q-*dependent self-scattering and Compton scattering components, as described by Drewitt *et al*. (*72*), where the scattering vector $q=\frac{4\pi }{\lambda }\text{sin}\theta$. The corresponding *G*(*r*) functions were obtained from the Fourier transform relation $(r)=\frac{1}{2\pi^{2}r\rho }\int_{0}^{q_{\text{max}}}q[S(Q)-1]\text{sin}(\mathrm{qr})dq$, where *r* is a distance in real space, ρ is the atomic number density, and *q*_max_ is the high-*q* truncation limit. Reproducibility of results was checked by repeating the fast cooling rate experiments two to three times, observing the same formation and/or resorption of nanolites in all cases.

### In situ SAXS-WAXS measurements

The crystal nucleation process in the melt was studied in situ by a synchrotron-based time-resolved simultaneous SAXS/WAXS technique at beamline I22 at the DLS, UK. High-temperature experiments were made using the same microheating cell (*69*) and methodology described in the XRD paragraph.

A monochromatic x-ray beam at 12.4 keV was used to obtain 2D scattered intensities, which were collected with Dectris Pilatus P3-2M large-area pixel array detectors at SAXS and WAXS geometries. The x-ray beam was focused down to 0.180 mm in the vertical and 0.480 mm in the horizontal dimensions. Data were acquired as series of 200-ms frames with a 500-ms dead time in between frames. The series were spaced in time by 1 s. Transmission was measured by a photodiode installed into the beamstop of the SAXS detector. A sample-to-SAXS detector distance was calibrated against a 100-nm periodicity grating, and a sample-to-WAXS detector distance was calibrated with a National Institute of Standards and Technology (NIST) silicon powder standard. The setup allowed for a usable *q* range of 0.03 < *q* < 31 nm^−1^ provided by SAXS and WAXS ranges overlap. The scattered intensity was calibrated to absolute units against a NIST glassy carbon standard. Different backgrounds were acquired from the empty wire heated to the temperature of interest before each experiment.

The initial SAXS data processing and reduction including masking, normalizations and correction for transmission, background subtraction, and data integration of the collected 2D data to 1D were performed with the DAWN software (*73*). Model fitting and validation were performed with use of the Small Angle Scattering Analysis Software Package SasView (*74*). An isometric (octahedral) crystal shape characterizes magnetite, and therefore, for modeling SAXS data, we approximated the shape of magnetite nanolites as spheres. SAXS patterns were modeled assuming a unimodal distribution of nanolites with the 1D scattering intensity calculated in the following way (*74*)

$$I(q)=\frac{\text{scale}}{V}\cdot {\left[3V(∆\rho )\cdot \frac{\text{sin}(\mathrm{qr})-\mathrm{qr}\;\text{cos}(\mathrm{qr})}{{\left(\mathrm{qr}\right)}^{3}}\right]}^{2}+\text{background}$$

where scale is a volume fraction, *V* is the volume of the scatterer, *r* is the radius of the sphere, background is the background level, and ∆ρ is the difference of the scattering length densities of the scatterer and the solvent (∆ρ = 40.7 × 10^−6^ was used for magnetite nanolites and ∆ρ = 25.6 × 10^−6^ for the melt).

### Magma analogs preparation and viscosity measurements

To characterize better the rheological effects of nanoparticles in magmas, we extended the range of previous investigations on analog microsuspensions (*26*, *64*) to a smaller particle size range by using SiO_2_ nanoparticles in silicon oil. This Newtonian fluid is commonly used to characterize the effect of microlites in magmas in terms of changes to the base fluid viscosity, volume percent, size, and shape of microparticles. Previous experiments have demonstrated how the increasing crystal content of a magma eventually causes the magma to “lock” as the viscosity increases exponentially to the fragmentation threshold. For microparticles, the critical volume percent ranges from 35 to 70 volume % depending on the crystal shape (*26*). This is referred to as the maximum packing density. As the volume fraction approaches the maximum packing fraction, the magma behaves as an increasingly non-Newtonian suspension, eventually fragmenting under applied deformation rates (*11*). For silica-rich magmas, the pure melt itself may begin with a viscosity of 10^6^ to 10^7^ Pa s, and so, fragmentation occurs (*11*, *13*) even before crystal growth. However, for low-silica, basaltic magma, the melt viscosity is more often closer to 10^1^ to 10^3^ Pa s, and so, a large volume percent of crystals would be required to elevate the bulk viscosity by four to six orders of magnitude (or an unreasonably high strain rate of 10,000 s^−1^) to reach explosive conditions.

The magma melt phase was represented by silicon oil, a Newtonian fluid with a viscosity of 0.136 Pa s (η_oil_) at 20°C. This is lower than the viscosity of pure basaltic melt at eruptive conditions (~200 Pa s) but allows a wider range of the volcanologically relevant parameter space to be investigated within the technical abilities of the rheometer. One advantage of using these analog mixtures is that the volume percent of particles can be very accurately controlled (at a constant temperature), and thereby, the effect of such particles on the viscosity can be accurately parameterized. As discussed in the section, Viscosity of magma analogs with nanoparticles, assuming the complete extraction of iron from the melt to form single crystal FeO nanolites, the maximum volume percent that could be formed would be ~7 volume %. We thus prepared nanoparticle-bearing suspensions containing ~few volume % of nanolites, as expected in nature. The shear rates used are those relevant to volcanological flows. The silicon oil was mixed with fumed SiO_2_ spherical nanoparticles with a particle average size of ~14 nm to obtain a suspension with a known volume percent of nanoparticles. The initial suspension was shaken and mixed horizontally overnight using an electronic roller mixer. Afterward, we centrifuged the mixture (silicon oil + nanoparticles) between 5 and 30 min at 4000 to 5000 rpm. This allowed the homogenization of the suspension and the removal of bubbles potentially entrapped during the preparation of the mixture. The suspensions were then subjected to ultrasonification ultrasound treatment to both maximize the uniform dispersion of nanoparticles and minimize particle aggregation in the base fluid. All suspensions displayed homogeneous colors, suggesting that samples were homogenized successfully after centrifuge and ultrasonification. However, to ensure complete homogenization, we gently stirred each sample before loading the suspension into the crucible for viscosity measurement. We performed the measurement of the density of the standard oil and suspensions at 25°C to retrieve the actual density of SiO_2_ nanoparticles and reveal any potential anomaly in the measure of the sample density due to the entrapment of air in the suspension. The calculated density of the fumed SiO_2_ particles was 1.9 ± 1 g cm^−3^ that agrees with the density of standard fumed SiO_2_. The density measurements were repeated weekly for 1 month, and no change in density with time was observed.

We used a MARS III (Modular Advanced Rheometer System) device equipped with a concentric cylinder geometry to measure the viscosity of suspensions. We loaded ~16 ml of sample into the crucible before each measurement. The viscosity was measured at 20°C according to the two-stage protocol. We applied a preshear treatment to the sample that involved shearing the suspension from 0 to 100 s^−1^ and then back again. This ensured removal of potential transient effects on the rheology of our sample due to particle reorientation and organization. Afterward, we performed viscosity measurement that consisted of three ramps where the shear rate was increased from 0 to 100 s^−1^ (upward ramp), kept constant at 100 s^−1^ for a minimum of 30 s (constant ramp), and then decreased from 100 to 0 s^−1^ (downward ramp). We stepped the shear rate value by ~3.3 s^−1^ through the upward and downward ramps to obtain 30 viscosity measurements during each segment. During each measurement, the stress was recorded until it reached a constant value. The viscosity measurements of our suspensions were repeated 2 weeks later, and no change in viscosity with time was observed.

### Simultaneous thermal analysis

The degassing and pumice formation experiments were performed using a simultaneous thermogravimetric analyzer-differential scanning calorimetry (TGA-DSC 3+, Mettler-Toledo, Switzerland) equipped with a water cooling device. The measurements were carried out under N_2_ atmosphere (60 ml min^−1^ flow rate) using a PtRh20 crucible.

## Supplementary Material

abb0413_SM.pdf

## SUPPLEMENTARY MATERIALS

Supplementary material for this article is available at http://advances.sciencemag.org/cgi/content/full/6/39/eabb0413/DC1
