# Supplementary material for: In situ observation of nanolite growth in volcanic melt: A driving force for explosive eruptions
Source: Sci Adv. 2020 Sep 23;6(39):eabb0413. doi: 10.1126/sciadv.abb0413 (PMC7531885; doi:10.1126/sciadv.abb0413)
Supplement: abb0413_SM.pdf [file abb0413_SM.pdf]

Science Advances
AAAS

advances.sciencemag.org/cgi/content/full/6/39/eabb0413/DC1

Supplementary Materials for

In situ observation of nanolite growth in volcanic melt: A driving force for explosive eruptions

Danilo Di Genova*, Richard A. Brooker, Heidy M. Mader, James W. E. Drewitt, Alessandro Longo, Joachim Deubener, Daniel R. Neuville, Sara Fanara, Olga Shebanova, Simone Anzellini, Fabio Arzilli, Emily C. Bamber, Louis Hennet, Giuseppe La Spina, Nobuyoshi Miyajima

*Corresponding author. Email: danilo.di-genova@uni-bayreuth.de

Published 23 September 2020, *Sci. Adv.* **6**, eabb0413 (2020)
DOI: 10.1126/sciadv.abb0413

The PDF file includes:

Sample descriptions for images in Figure 1
In situ XRD measurements
In situ SAXS-WAXS measurements
Modelling of nanoparticle agglomeration
Simultaneous Thermal Analysis (STA)
The time dependence of the nanolite viscosity effect
Appendix: Relationships used in agglomeration modelling
Figs. S1 to S10
References

Supplementary Materials

Sample descriptions for images in Figure 1

The Colli Albani scoria is K-Foidite scoria from the 41±9 ka Albano 7 sub-Plinian eruption (*42*) in the ultrapotassic Roman Province (Colli Albano volcano, Italy). Preliminary analysis (STEM-EDAX) suggest the nanolites are Fe-rich (i.e. magnetite). The Tambora sample is a pumice from the Plinian stage of the 1815 eruption [TB-065 of (*25*)]. The spherical nanolites are Fe-rich but the lath-shaped crystals are K-bearing (possibly biotite). Although not actual 'basalts', the Colli Albani and Tambora compositions both have low calculated viscosities, similar to a typical basalt, of ~10^2 Pa s at eruption temperatures due to their low silica content, with Colli Albani melt containing ~2.5 wt % water at 1150°C and Tambora containing 3 wt % water at 1000°C. This viscosity well below the minimum fragmentation threshold of ~10^6 Pa s required for an explosive eruption at realistic shear rates (*41*). Tambora samples (*25*) have an average crystal content of ~20 volume % [present as mm-sized phenocrysts]. About 30 polished sections were examined representing 24 pumice clasts (some were orthogonal sections) the vast majority being grey pumice clasts from the main F4/5 Plinian phase of eruption (*25*). Some are from the collection in Sigurdsson and Carey (*25*), the others were collected by Jessica Kandlbauer and Steve Sparks. The phenocrysts in Tambora grey pumice are generally equant. The maximum sectioned aspect ratio for a few clinopyroxene crystals is 1:8, whereas the plagioclase is always much less, typically 1:3. However, the crystals tend to form intergrowth clots of 3-5 crystals which are more 'equant' than the individual crystals. Of the grey pumice sections analysed, the 2D crystal content ranged from 10 to 30 % of the area of the glass, the majority around 20%. The Colli Albani crystal content is less than 40 volume % (*18*). The Mt. Etna 122 BCE sample comes from the early phase of the Plinian eruption (C3) and has a few subhedral iron oxides as well as the much more common features comprised of smaller particles, apparently agglomerated into 50 nm spheres, that are similarly Fe-rich (Fig. 1f). These nanolites are present in the interstitial melt between numerous microlites. Sable et al. (*14*) looked at other units in the Mt. Etna 122 BCE eruption and suggested interstitial melt could be as high as 57 volume % between microlites (between 1 and ~100 μm) although many samples have less melt (our C3 has ~50 volume %). However, they also noted that, at high magnification, the glassy matrix could "appear mottled and seems to be a collection of tiny crystallites with poorly resolved boundaries". The hydrous samples (*47*, *76*) from experiments illustrate how nanolites can be grown in both high pressure wet melts and quenched glasses.

The results of our observations show that the glassy matrix of the products erupted during the Mt. Etna 122 BCE Plinian eruption and the sub-Plinian eruption of Colli Albani is homogeneously permeated by nanolites. For samples from Tambora, we observed a variation in the nanolite peak intensity at 670 cm^{-1}, which tended to be higher close to crystals. In Fig. 1a we report the two endmembers of Raman spectra. This change in intensity suggests that the samples have a heterogeneous number density and size of nanolites as discussed for the two Mt. Etna hydrous glasses subjected to STA experiment (see the main text and "Simultaneous Thermal Analysis (STA)" paragraph in Supplementary Materials section). For the case study

of Mt. Etna 122 BCE, we sampled different sizes (from ~1 to ~10 cm) of clasts of the Plinian phase. Furthermore, we also studied scoria erupted at Mt. Etna during a weak explosive eruption in 2011. The samples from the 2011 eruption were also subjected to SEM, TEM measurements and Raman spectroscopy analysis. Nanolites were not found.

The nanolite identification strategy was initially based on the use of Raman spectroscopy to map the presence of nanolites thanks to the relatively large spatial resolution of the analytical technique. We subsequently carried out TEM analyses on the sample areas identified by the Raman spectroscopy analysis. We observed neither a spatial nor a size distribution of nanolites from the edge (fast quench) towards the centre (slow quench) of samples. We thus exclude that post-fragmentation quench of clasts as a mechanism responsible for nanolite formation. The Mt. Etna experimental samples of Fig. 2 and Fig. 3 were subjected to Raman spectroscopy measurements after XRD-SAXS-WAXS measurement at the synchrotron to determine the spatial distribution of the nanolite. We did not find any variation in the Raman spectrum along the sample.

In situ XRD measurements

In fig. S1 the XRD scattering intensity for the pure Mt. Etna basalt melt at 1300°C (above the liquidus), and the glass when quenched to 25°C from 1300°C at the maximum possible quench rate (turning the furnace off), both in air, are shown in fig. S1a. The coherent reciprocal-space scattered intensity for the liquid and glass represented by the total structure factors $S(q)$ are shown in fig. S1b. The corresponding real-space pair distribution $G(r)$ functions shown in fig. S1c provides a measure of probability of finding two atoms a distance r apart. The liquid $S(q)$ exhibits diffuse first and second peaks at 2.05 and 4.8 Å^{-1}. On quenching to a glass, these peaks become slightly more pronounced, indicating an increase in atomic-scale ordering. In real-space, the first peak in $G(r)$ at ~1.6 Å in both the liquid and glass arises from the nearest-neighbour T-O bond in tetrahedral units (where T is Si^{4+}, Al^{3+}, Fe^{3+}). A second peak at 3.06 Å corresponds to the Si-Si bond length, while the peak at 4.08 Å is attributed to the second nearest-neighbour Si-O bond. A small feature appearing in the glass $G(r)$ at ~ 2.5 Å is consistent with O-O correlations becoming more pronounced on vitrification. Due to the complexity of the sample composition, no other cation-O or cation-cation correlations are resolved.

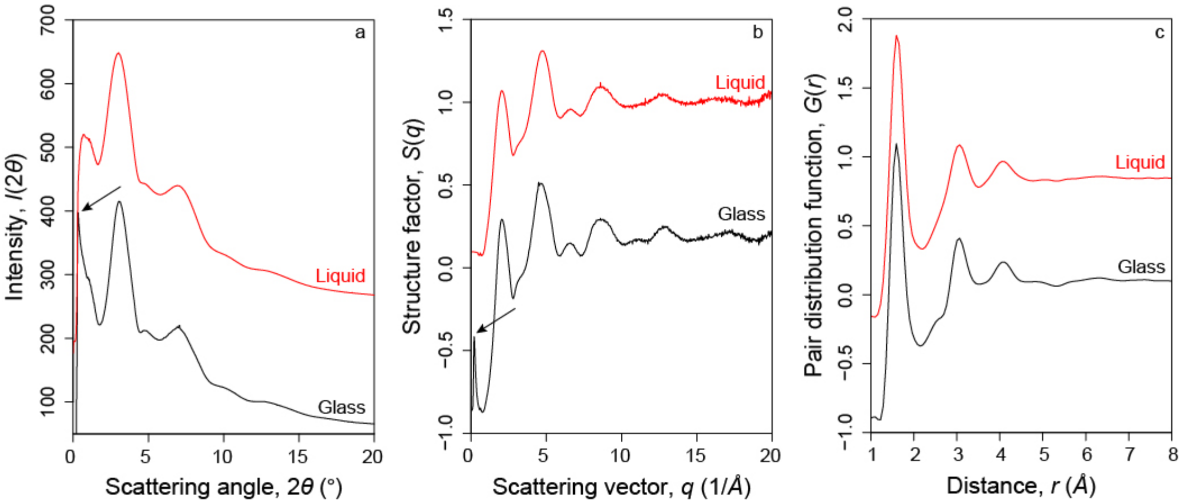

Figure S1. Results of *in situ* XRD measurements. **(a)** Diffraction patterns obtained for the liquid basalt at 1300°C and rapidly quenched glass at 25°C, **(b)** the liquid and glass structure factors, $S(q)$, and **(c)** the corresponding pair distribution $G(r)$ functions, as obtained by Fourier

transforming the corresponding structure factors in (b). The arrows indicate the position of the small angle nanolite peak. For clarity, the glass data have been displaced vertically.

The rapidly-quenched glass also exhibits a sharp diffraction peak at 0.3° (0.2 $Å^{-1}$), indicated by the black arrows in fig. S1a and fig. S1b. The peak is truncated at low scattering angles by the shadow of the tungsten beam-stop. The characteristically glassy structure factor combined with the sharp very-small-angle scattering peak is consistent with coexistence between a glass matrix and 'particles' on the order of nanometers in size (*45*). Unfortunately, the peak is truncated at low scattering angles below 0.3° (0.2 $Å^{-1}$) by the shadow of the tungsten beam-stop (see fig. S1 and Fig. 2). This precludes full exploitation of this small angle peak that is potentially a rich source of information.

In situ SAXS-WAXS measurements

The evolution with time of the SAXS pattern reported in Fig. 3c suggests an increase in both the particle size and interaction. A 'hard sphere model' was used to describe the SAXS patterns. This model calculates the interparticle structure factor for monodisperse spherical particles interacting through hard sphere (excluded volume) interactions. Parameters involved in the model are the effective hard sphere radius and the volume percent occupied by the spheres. After 65 seconds into the 950°C isothermal crystallization event the volume percent occupied by the first population of spheres only is estimated to be about 25 volume %. The second local maxima due to a second population of nanolites was fitted independently as a unimodal distribution of hard spheres.

For the initial XRD collection the resolution was optimised for a liquid diffraction pattern so the q step size is too large to resolve small peaks that might arise from the nanolites in Fig. 2b. Therefore, it is difficult to know if these features are crystalline, immiscible melts, or heterogeneities in the melt structure. However, the WAXS data shown in Fig. 3b, with higher q-space resolution, clearly demonstrates these are crystalline features. The Bragg peak intensity increases slowly between 15 and 23 s, whereas afterwards we observed a faster increase and the appearance of new peaks at 2.128, 2.316, 2.47, 2.48, 2.51, 2.554, 2.806, 3.004, 3.173, 3.634, 4.135 and 4.144 $Å^{-1}$.

This again demonstrates the fast development of these features at these near-liquidus temperatures. The nanolite peak height at 890°C is the same as the quenched sample suggesting all the growth occurs above this temperature with little more happening down to the glass transition temperature at 700°C. Applied to a natural eruption, this clearly demonstrates that nanolites can grow much more quickly than microlites and are a feature of rapid undercooling. The natural nanolites shown in Fig. 1 are, therefore, the result of rapid growth and are more likely to result from a dynamic pre-eruptive environment than the rapid quenching produced post-fragmentation as magma is ejected.

Modelling of nano-particle agglomeration

The idea here is that agglomerates of particles entrap and immobilise liquid, effectively increasing the volume percent of solids. In other words, the volume of an agglomerate is larger than the sum of the particle volumes. Agglomerates can rapidly grow in volume as the particle concentration increases (*77-78*) and/or shearing (*36*) promoting the observed early onset of non-Newtonian behaviour at extremely low volume fraction ϕ and the dramatic increase in the bulk viscosity observed in Fig. 4. We here consider the volume of agglomerates and whether we can thereby account for the observed rheology. The approach is entirely geometrical. The calculations below allow us to gauge the scale of liquid entrapment and hence whether it might feasibly explain the rheology.

For simplicity, we consider agglomerates of spherical particles with uniform radius r. This is entirely appropriate for our experimental suspensions, as the particles used were

spherical. We assume furthermore that agglomerates are formed in a fcc close-packed arrangement (fig. S2).

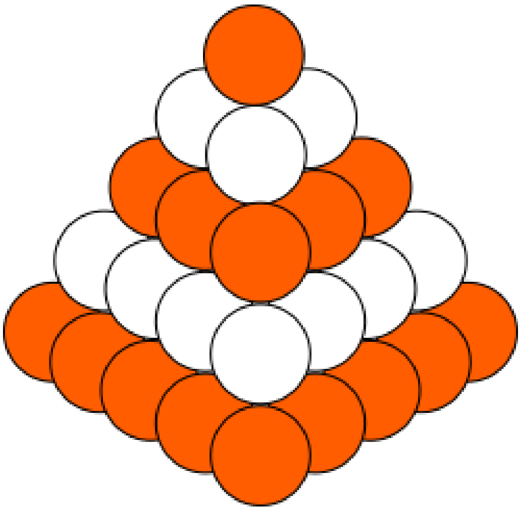

Figure S2. Face-centred-cubic (fcc) packing showing 5 layers.

In general, a stack of m layers will contain n particles:

$$n = \binom{m+2}{3} = \frac{(m+2)!}{3!\,(m-1)!} = \frac{m(m+1)(m+2)}{6} \qquad \text{(S1)}$$

The smallest such agglomerate involves 2 layers with 4 identical spherical particles in a tetrahedral arrangement such that the centre of each particle sits on one vertex of a tetrahedron (fig. S3a). Each particle is surrounded by a film of immobile liquid of thickness $\delta = \alpha r$ where $\alpha \geq 0$ gives the film thickness as a fraction of the particle radius r. The radius of particle plus the liquid film is then:

$$R = r + \alpha r = (1 + \alpha)r \qquad \text{(S2)}$$

Moreover we assume that liquid trapped between the particles is immobile. For the smallest agglomerate of just 4 particles, each face will look as shown in fig. S3b. The 4th particle will sit on top of these 3 particles. The green colour shows the immobile liquid.

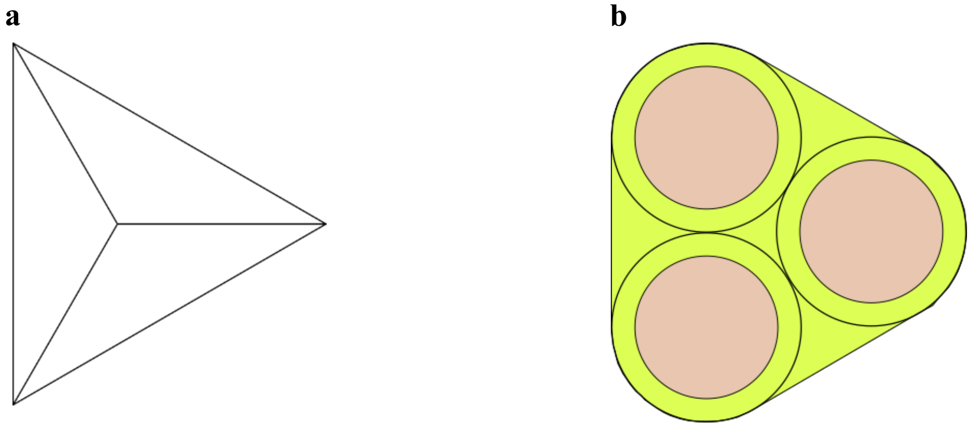

Figure S3. (a) Regular tetrahedron. All four faces are equilateral triangles of the same size. **(b)** A face of a tetrahedrally-coordinated agglomerate of 4 particles with the spheres shown in projection onto the face. The green colour shows the liquid. The 4th particle sits centrally on top of these particles.

In order to work out the volume of particles plus entrapped liquid, we must first work out the volume of the smallest tetrahedron that encloses the particles. Fig. S4 shows the base of a 2-layer stack of 4 particles. The 3 particles in the base layer are in contact with the tetrahedron's basal face a perpendicular distance R below their centre points A, B and C. The 4th particle is placed above these 3 particles (see figs S2 and S3b).

Consider the tetrahedron that contains only the centres of all the spheres in the m layers. In the 2-layer system shown in fig. S3b the base of this tetrahedron is given by the triangle ABC. In the general case with m layers, the edge length $\overline{AB}$ of this tetrahedron containing only the centres of the spheres is:

$$a' = 2R(m-1) \tag{S3}$$

(i.e. just $2R$ in fig. S4 as $m = 2$) and its height is (see Appendix Equation A7):

$$h' = \frac{\sqrt{6}}{3}a' = \frac{\sqrt{6}}{3}2R(m-1)\ . \tag{S4}$$

We now need to move all sides outwards by R so that the spheres will be fully contained. The perpendicular distance from the centre of the volume to the face is $cf' = h'/4$ (see Appendix Equation A12). This becomes $cf = h'/4 + R$ after shifting and hence the tetrahedron fully enclosing the spheres has a height of:

$$h = h' + 4R = \left[\frac{\sqrt{6}}{3}2(m-1) + 4\right]R \tag{S5}$$

and an edge length of:

$$a = \frac{3}{\sqrt{6}}h = 2R(m-1) + 2\sqrt{6}R \tag{S6}$$

Furthermore, we can see that:

$$b = \frac{a - 2R(m-1)}{2} = \sqrt{6}R \tag{S7}$$

and that the length $\overline{OB}$, which is the perpendicular distance from the centre of one of the spheres to an edge, is given by:

$$\overline{OB} = b \times \sin 30 = \frac{\sqrt{6}}{2}R = 1.22R \tag{S8}$$

The base extends beyond particles in all directions because the faces are not perpendicular to the base. The faces touch the spheres tangentially some distance above the base.

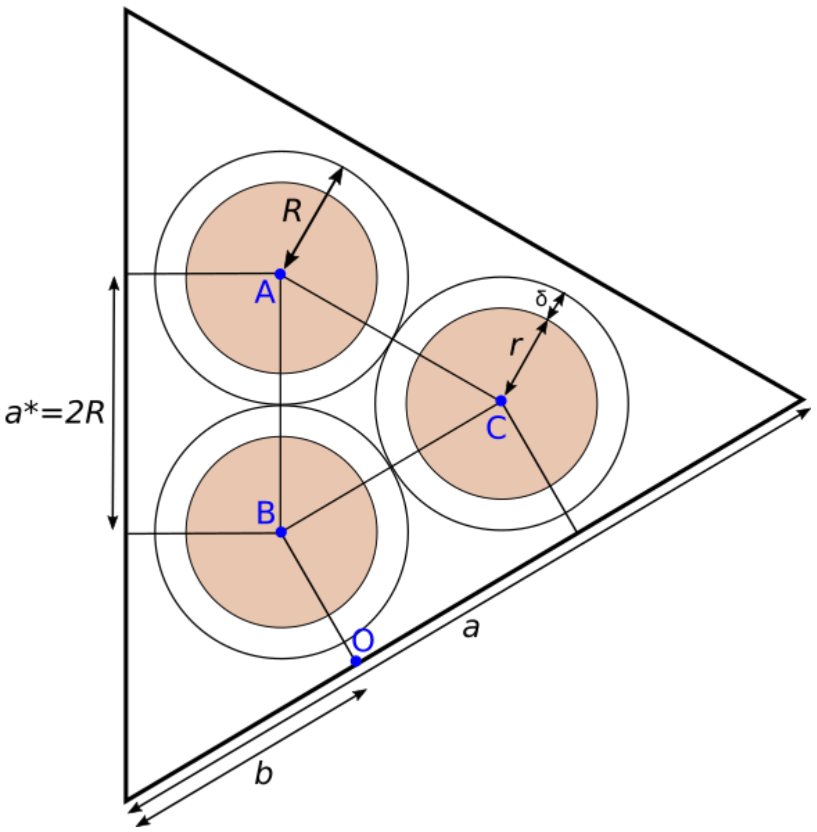

Figure S4. The basal plane of a regular tetrahedron enclosing 4 particles. The particles shown touch the base tangentially a radial distance below the points A, B and C. The particles are shown in projection onto the basal plane. $\delta = \alpha r$ see Equation (S2).

The volume of the tetrahedron is:

$$V_a = \frac{a^3}{6\sqrt{2}} = \frac{\left[2(m-1)+2\sqrt{6}\right]^2}{6\sqrt{2}} R^3 \qquad \text{(S9)}$$

However this volume is larger than the volume shown in fig. S3b because of the excess volume beyond the spheres along the edges and in the vertices. Consider first the excess volume in the vertices. We can work out this volume by calculating the volume of a tetrahedron that encloses a single particle (i.e. we have a single layer so $m = 1$). Subtracting the volume of the sphere gives us the excess volume in at vertices.

For $m = 1$, from Equation (S6) we get:

$$a_1 = 2\sqrt{6}R = 4.899R \qquad \text{(S10)}$$

The volume of the tetrahedron is then using Equation (S9):

$$V_1 = 8\sqrt{3}R^3 = 13.856R^3 \qquad \text{(S11)}$$

The excess volume at the vertices is then:

$$V_v = V_1 - \frac{4}{3}\pi R^3 = 9.668R^3 \qquad \text{(S12)}$$

Note that V_v is independent of the number of layers m as there are always 4 vertices and they always have the same volume. Consequently, this excess volume is appreciable only for small agglomerates. For our limiting case of a single particle, the excess volume is more than twice the volume of the sphere itself.

Now consider the excess volume along the edges. This volume consists of the grey area shown in fig. S4 extending along the edge perpendicular to the page (refer to fig. S5 and Equation A14 in the Appendix).

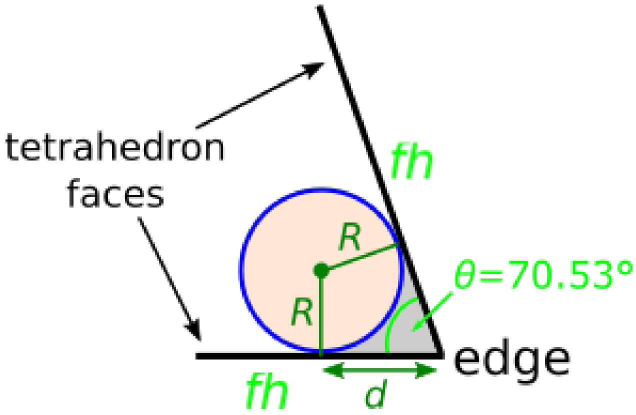

Figure S5. Sphere between two faces of the tetrahedron. The tetrahedron edge is perpendicular to the page. The excess liquid is indicated in grey.

The grey area in fig. S5 is given by working out the area of the quadrilateral into the edge and then removing the circle sector bounded by the two radii shown:

$$A_e = Rd - \frac{\theta R^2}{2} = \left(\frac{1}{\tan\frac{\theta}{2}} - \frac{\pi-\theta}{2}\right)R^2 = 0.459R^2 \qquad \text{(S13)}$$

This extends along the edge for the distance between the centres of the spheres $2R(m-1)$. So the excess volume along an edge is:

$$V_e = 0.918(m-1)R^3 \qquad \text{(S14)}$$

The total volume of the agglomerate plus liquid is then, using equations (S9), (S12) and (S14):

$$V = V_a - V_v - 6V_e \qquad \text{(S15)}$$

$$V = \left[\frac{\left(2(m-1)+2\sqrt{6}\right)^3}{6\sqrt{2}} - 9.668 - 5.508(m-1)\right]R^3 \qquad \text{(S16)}$$

For our smallest agglomerate with 2 layers ($m = 2$) and 4 spheres ($n = 4$) Equation (S16) reduces to:

$$V_2 = (38.698 - 9.668 - 5.508)R^3 = 23.522R^3 \qquad \text{(S17)}$$

The 4 spheres have a volume of $n \times \frac{4}{3}\pi R^3 = 16.755R^3$ so the agglomerate is ~40% larger in volume.

For an agglomerate with 3 layers ($m = 3$) and 10 spheres ($n = 10$) we get:

$$V_3 = (83.053 - 9.668 - 11.016)R^3 = 62.369R^3 \quad \text{(S18)}$$

which is ~50% increase in volume.

The above calculations for 2 and 3 layers assume no additional thin film around the particles, i.e. $\alpha = 0$ and so $r = R$ in Equation (S2) above. To work out the film thickness (i.e. α as defined in equation S2) we need to explain the rheology, we need first to determine what volume fraction of (micro) particles would produce the observed behaviour. We can use the following graph (fig. S6) from Mader et al. (*26*).

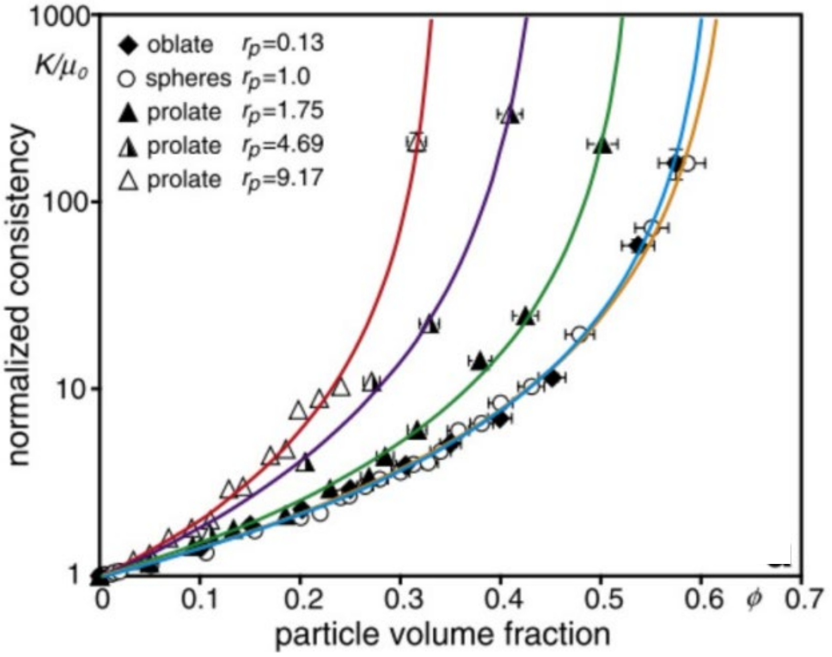

Figure S6. Graph of normalized consistency (equivalent to relative viscosity) as a function of particle volume fraction for particles with different aspect ratio r_p (*26*).

At $\phi = 0.04$ of nano-particles, the viscosity is increased by a factor of 100. This would be at $\phi = 0.6$ for micro-particles (see the yellow line for spheres in fig. S5). This is an increase of a factor of 15 in volume fraction. In other words, we need to find the value of α such that the agglomerate volume given by Equation (S16) is $15 \times\ n \times \frac{4}{3}\pi R^3$.

We write the volume of the agglomerate as:

$$V = KR^3 = K(1+\alpha)^3 r^3 \quad \text{(S19)}$$

where $K = \frac{\left(2(m-1)+2\sqrt{6}\right)^3}{6\sqrt{2}} - 9.668 - 5.508(m-1)$ from equation S16.
The condition on α we seek is then:

$$K(1+\alpha)^3 r^3 = 15n\frac{4}{3}\pi r^3 \quad \text{(S20)}$$

and hence:

$$\alpha = \sqrt[3]{62.832\frac{n}{K}} - 1 \quad \text{(S21)}$$

For 2 layers ($m = 2$) and 4 spheres ($n = 4$) (see Equation S17) $K = 23.522$ and we get:

$$\alpha_2 = 1.20 \quad \text{(S22)}$$

For 3 layers ($m = 3$) and 10 spheres ($n = 10$) (see Equation S18) $K = 62.369$ and we get:

$$\alpha_3 = 1.16 \quad \text{(S23)}$$

The graph in fig. S7 shows that α grows rapidly with agglomerate size, given by the number of layers m. Note that the point at $m = 1$ is for a single particle, so not really an agglomerate. α reaches a stable value of just over ~1 for agglomerates with ≥ 2 layers (i.e. 4 particles or more). In other words, we would need a thickness of immobile liquid around each particle of around $\sim r$ (a particle radius – see Equation S2 for the definition of r).

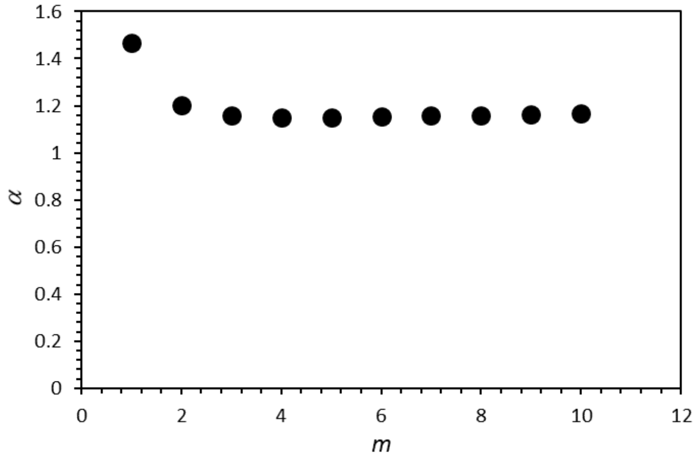

Figure S7. Graph of the film thickness α (film thickness as fraction of particle radius) as a function of m (numbers of layers).

Simultaneous Thermal Analysis (STA)

Figures S8a, b show optical microscope images of the nanolite-bearing basaltic pumice in Fig. 5b (STA Experiment 1). Note the high number of micrometric bubbles. Figure S8c shows Raman spectra of the nanolite-bearing basaltic pumice (STA Experiment 1, Fig. 5a) and black and dense glass recovered after the STA Experiment 2 that was stopped at 620°C. The signature at ~670 cm^{-1}, similar to that in Fig. 1, arises from FeO-bearing nanolites dispersed in the glass matrix (*30*), whilst the contribution at ~930 cm^{-1} arises from the Fe^{3+} in the glass structure (*79*). The peak at ~670 cm^{-1} is more developed in the Raman spectrum of the basaltic-pumice (STA Experiment 1). On the other hand, the contribution at ~930 cm^{-1} is much more developed in the Raman spectra collected from visually-unaffected sample from the STA Experiment 2, suggesting that most of the iron is still-structurally bonded to the glass structure. Interpretations of Raman spectra agree with observations (i.e. size and relative number density of nanolites, see main text for a discussion) derived from HAADF-STEM and STEM-EDS analyses of the two samples (Figs. 5c, d, e, f, g).

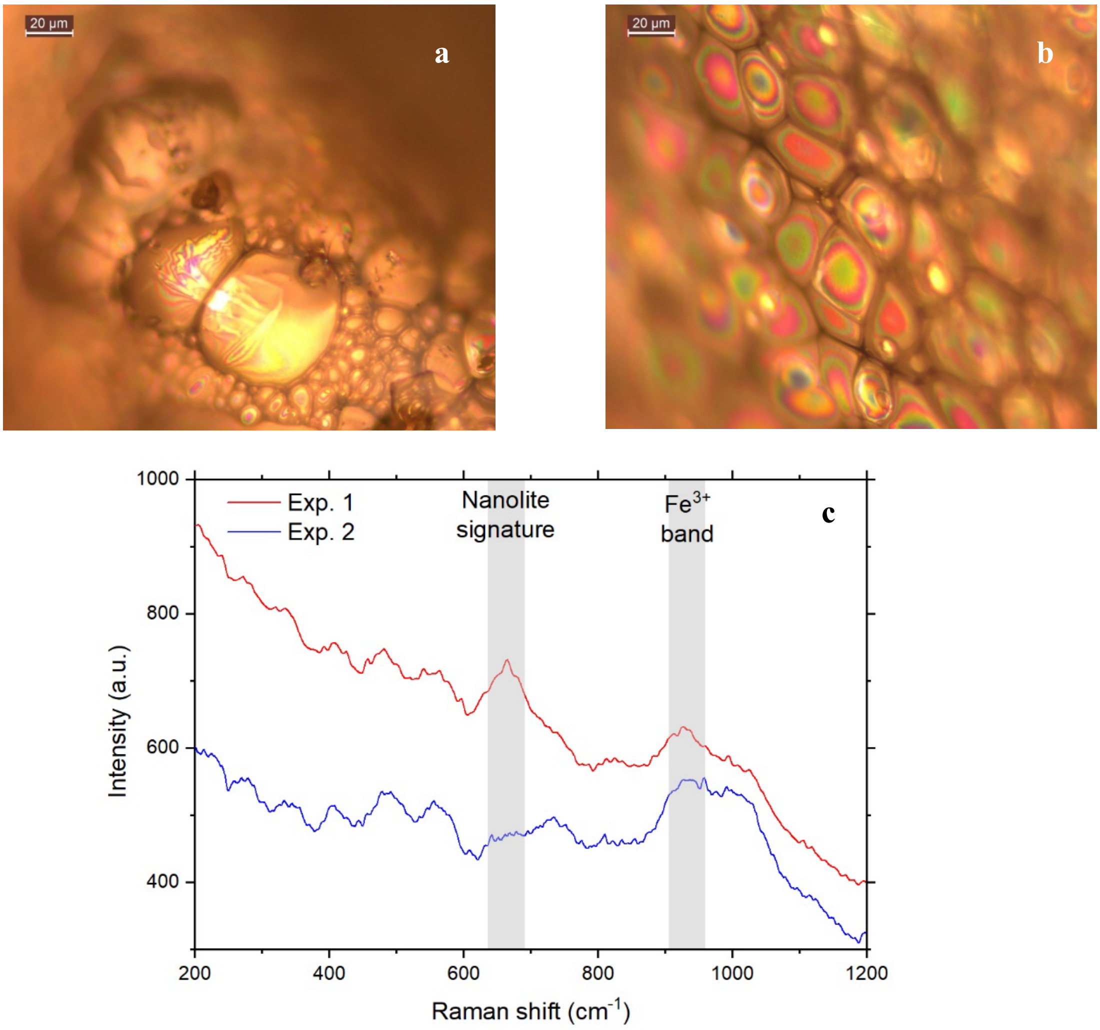

Figure S8. (a, b) Optical microscope images of the nanolite-bearing pumice (Fig. 5b) obtained with the STA Experiment 1 (Fig. 5a) using a dense and synthetic hydrous basaltic glass (H_2O = 1.48 wt.%) with a Mt. Etna composition. HAADF-STEM and STEM-EDS images of this sample are shown in Figs. 5c, d, e. **(c)** Raman spectra representative of STA Experiment 1 (Fig. 5a); the nanolite-bearing basaltic pumice (figs. 5b, c, d, e and figs. S8a, b), and STA Experiment 2 that was stopped at 620°C, where the sample recovered at the end of the STA Experiment 2 was visually unaffected with respect to its initial state (i.e., a dense black glass). See HAADF-STEM and STEM-EDS images in figs. 5f, g.

The time dependence of the nanolite viscosity effect

Figure S9a shows the relationship between the maximum packing fraction (ϕ_m) and the particle size obtained combining our results from rheological measurements of nano-suspensions and those from Del Gaudio et al. (*64*) who used micro-suspensions. The maximum packing fraction decreases significantly with decreasing particle size, reflecting the size-dependence of the particle agglomeration process, namely the interplay between the particle size, their interaction, and the size of the immobile layer α (see “Modelling of nano-particle agglomeration” section above).

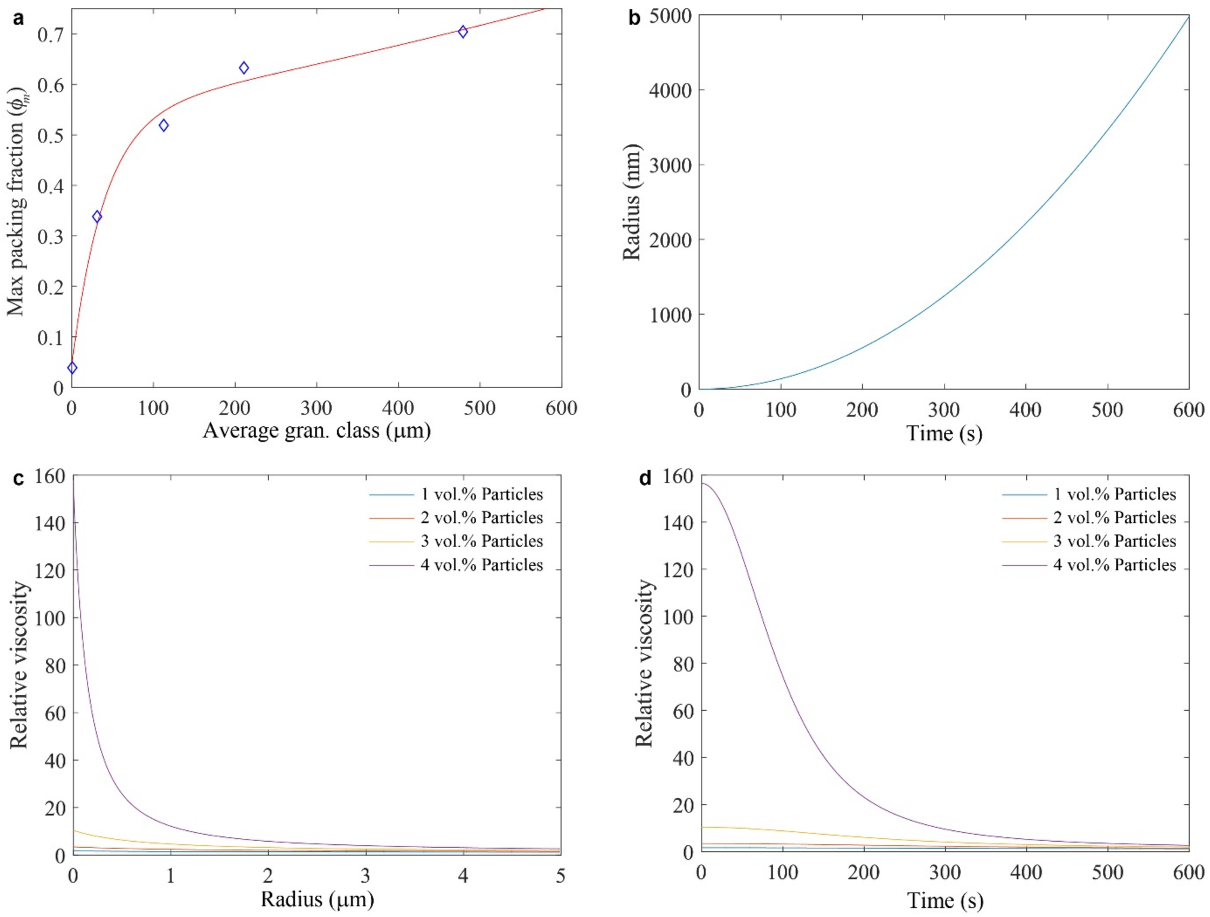

Figure S9. (a) Following Del Gaudio et al. (*64*), the maximum packing fraction is fitted as a function of the average granulometric class (i.e. particle size). In addition to the data from Del Gaudio et al. (*64*), we calculated the maximum packing fraction (ϕ_m) of our nano-suspension (ϕ_m = 0.045 and average gran. class = 0.015 mm, Fig. 4a). **(b)** Extrapolated evolution (using an exponential law) of the radius of nanolites over time using data reported in Fig. 3c. **(c, d)** Using the relative viscosity algorithm reported in Mader et al. [(*26*); see their Fig. 14], the relative viscosity is plotted as function of particle size (c) and of time (d), assuming the relationship between particle size and the maximum packing fraction (a) and the timescale of particle growth (b).

Figure S9b shows the evolution of the nanolite radius with time (first population of nanolites appearing) by fitting our data in Fig. 3c with an exponential law. The combination of dependence of ϕ_m as a function of particle size (fig. S9a) with the evolution of the nanolite size with time (fig. S9b) allows us to describe and extrapolate ϕ_m through time.

Figures S9c, d show, for different volume % of particles, the relationship between the relative viscosity of the suspension as a function of particle size and time, respectively. In particular, fig. S9c shows the dramatic increase in relative viscosity for just 4 volume % of particles with reducing particle size (i.e. from micro to nano) which reflects the equally dramatic reduction in maximum packing with reducing particle size shown in fig. S9a. Figure S9d shows the rapid reduction in viscosity with time as the particles grow. Initially, when the particles are in the nano-size range, the viscosity is very large because the maximum packing fraction is very low. As the particles grow into the micro-size range this effect vanishes and we reach the conventional regime where the viscosity increase is controlled primarily by volume percent rather than particle size.

Appendix: Relationships used in agglomeration modelling

This section summarises some of the geometrical relationships of equilateral triangles and regular tetrahedra used in the previous section 'Modelling of nano-particle agglomeration'.

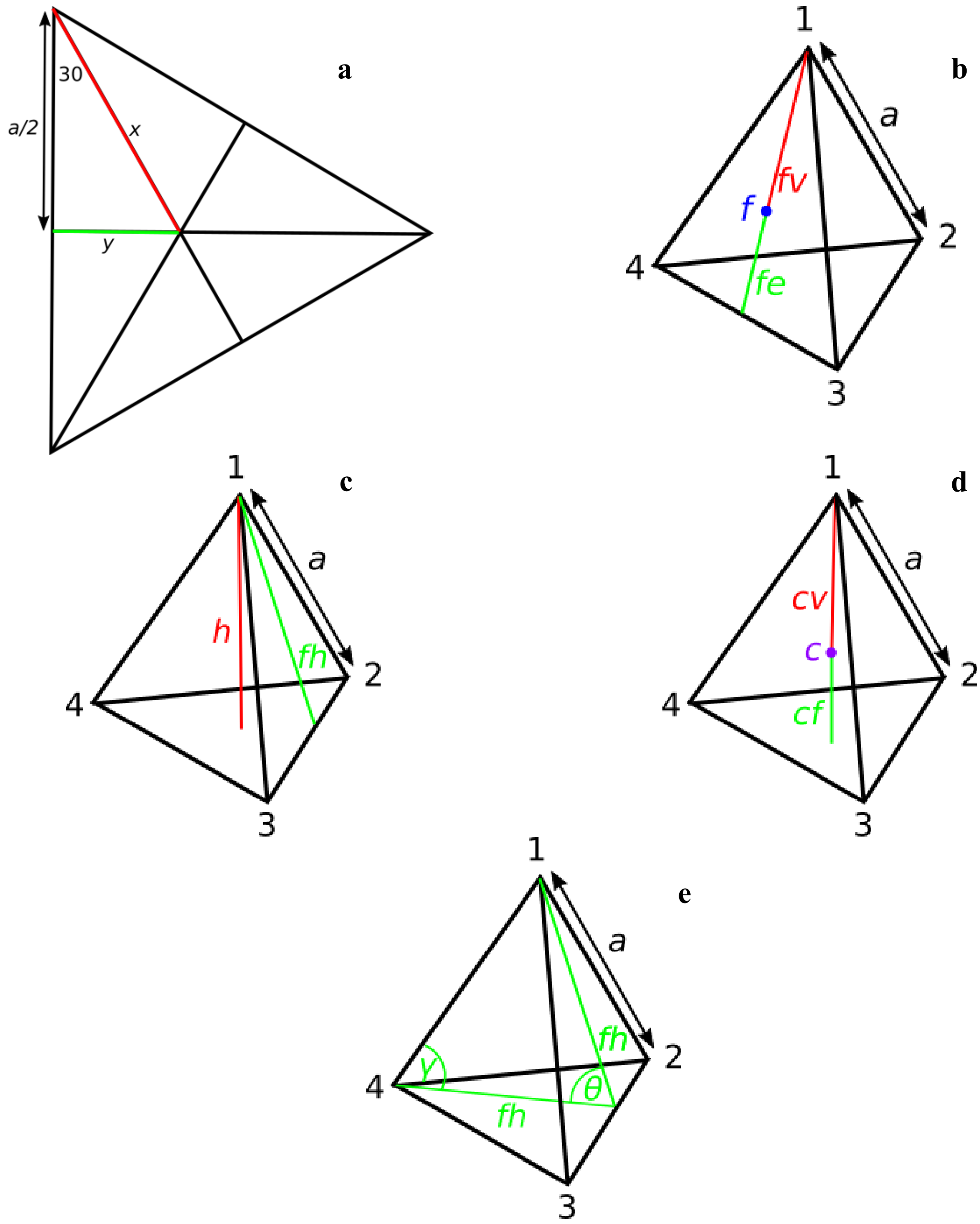

Figure S10. (a) Equilateral triangle. **(b)** Regular tetrahedron with side a showing centre of face and distances to vertex and edge. **(c)** Regular tetrahedron showing height of tetrahedron h and height of face fh. **(d)** Regular tetrahedron showing centre of volume and distances to vertex and face. **(e)** Regular tetrahedron showing angle between faces and angle of edge to the opposing face.

Relationships in equilateral triangles (fig. S10a):

$$x = \frac{a/2}{cos30} = \frac{a}{\sqrt{3}} \tag{A1}$$

$$y = xsin30 = \frac{a}{2\sqrt{3}} \tag{A2}$$

The height of the triangle is then:

$$h_t = x + y = \frac{\sqrt{3}}{2}a \tag{A3}$$

and the area:

$$A = \frac{1}{2}ah_t = \frac{\sqrt{3}}{4}a^2 \tag{A4}$$

Useful surds: $sin30 = \frac{1}{2}$ $cos30 = \frac{\sqrt{3}}{2}$ $tan30 = \frac{1}{\sqrt{3}}$

The distance from centre of face to vertex (fig. S10b):

$$fv = x = \frac{a}{\sqrt{3}} \tag{A5}$$

distance from centre of face to edge:

$$fe = y = \frac{a}{2\sqrt{3}} \tag{A6}$$

Height of tetrahedron (fig. S10c):

$$h = \sqrt{a^2 - x^2} = \sqrt{\frac{2}{3}}a = \frac{\sqrt{6}}{3}a \tag{A7}$$

Height of face:

$$fh = fv + fe = \frac{\sqrt{3}}{2}a \tag{A8}$$

Volume of tetrahedron:

$$V = \frac{1}{3} \times A \times h = \frac{a}{6\sqrt{2}} \tag{A9}$$

where A is the area of one of the faces (see Equation A4).

Angle between fh and h (fig. S10d, e):

$$cos\alpha = \frac{h}{fh} = \frac{2\sqrt{2}}{3} \quad \text{(A10)}$$

Distance from centre of volume to vertex:

$$cv = fv\ cos\alpha = \frac{\sqrt{3}}{2\sqrt{2}} a \quad \text{(A11)}$$

distance from centre of volume to face:

$$cf = h - cv = \frac{1}{2\sqrt{6}} a = \frac{h}{4} \quad \text{(A12)}$$

We can also work out several important angles.

The angle between faces can be worked out from the cosine rule:

$$fh^2 + fh^2 - 2\,fh^2\,cos\theta = a^2 \quad \text{(A13)}$$

Using Equation A8, we get $cos\theta = \frac{1}{3}$ and hence:

$$\theta = 70.53° = 0.392\pi \quad \text{(A14)}$$

Similarly, the angle of the edge to the opposing face is:

$$fh^2 + a^2 - 2\,fh\,a\,cos\gamma = fh^2 \quad \text{(A15)}$$

From which:

$$\gamma = 54.74° = 0.304\pi \quad \text{(A16)}$$

REFERENCES AND NOTES

1. P. W. Lipman, D. R. Mullineaux, The 1980 eruptions of Mount St. Helens, Washington (1981); 10.3133/pp1250.

2. C. G. Newhall, R. S. Punongbayan, The Narrow Margin of Successful Volcanic-Risk Mitigation, in *Monitoring and Mitigation of Volcano Hazards* (Springer Berlin Heidelberg, 1996), pp. 807–838.

3. P. Brooker, Fear in a handful of dust: Aviation and the Icelandic volcano. *Significance* **7**, 112–115 (2010).

4. B. Langmann, A. Folch, M. Hensch, V. Matthias, Volcanic ash over Europe during the eruption of Eyjafjallajökull on Iceland, April-May 2010. *Atmos. Environ.* **48**, 1–8 (2012).

5. J. Rougier, R. S. J. Sparks, L. Hill, *Risk and Uncertainty Assessment for Natural Hazards* (Cambridge Univ. Press, 2013).

6. N. F. Jones, Pliny the Younger's Vesuvius "Letters" (6.16 and 6.20). *Class. World.* **95**, 31–48 (2001).

7. C. Oppenheimer, Climatic, environmental and human con sequences of the largest known historic eruption: Tambora volcano (Indonesia) 1815. *Prog. Phys. Geogr.* **27**, 230–259 (2003).

8. D. M. Pyle, Forecasting sizes and repose times of future extreme volcanic events. *Geology* **26**, 367–370 (1998).

9. H. M. Gonnermann, M. Manga, The fluid mechanics inside a volcano. *Annu. Rev. Fluid Mech.* **39**, 321–356 (2007).

10. H. J. Hui, Y. Zhang, Toward a general viscosity equation for natural anhydrous and hydrous silicate melts. *Geochim. Cosmochim. Acta* **71**, 403–416 (2007).

11. P. Papale, Strain-induced magma fragmentation in explosive eruptions. *Nature* **397**, 425–428 (1999).

12. Y. Zhang, A criterion for the fragmentation of bubbly magma based on brittle failure theory. *Nature* **402**, 648–650 (1999).

13. A. Namiki, M. Manga, Transition between fragmentation and permeable outgassing of low viscosity magmas. *J. Volcanol. Geotherm. Res.* **169**, 48–60 (2008).

14. J. E. Sable, B. F. Houghton, P. Del Carlo, M. Coltelli, Changing conditions of magma ascent and fragmentation during the Etna 122 BC basaltic Plinian eruption: Evidence from clast microtextures. *J. Volcanol. Geotherm. Res.* **158**, 333–354 (2006).

15. M. Coltelli, P. Del Carlo, L. Vezzoli, Discovery of a Plinian basaltic eruption of Roman age at Etna volcano, Italy. *Geology* **26**, 1095–1098 (1998).

16. G. P. L. Walker, S. Self, L. Wilson, Tarawera 1886, New Zealand — A basaltic plinian fissure eruption. *J. Volcanol. Geotherm. Res.* **21**, 61–78 (1984).

17. B. F. Houghton, C. J. N. Wilson, P. Del Carlo, M. Coltelli, J. E. Sable, R. Carey, The influence of conduit processes on changes in style of basaltic Plinian eruptions: Tarawera 1886 and Etna 122 BC. *J. Volcanol. Geotherm. Res.* **137**, 1–14 (2004).

18. S. Campagnola, A. Vona, C. Romano, G. Giordano, Crystallization kinetics and rheology of leucite-bearing tephriphonolite magmas from the Colli Albani volcano (Italy). *Chem. Geol.* **424**, 12–29 (2016).

19. M. Pompilio, A. Bertagnini, P. Del Carlo, A. Di Roberto, Magma dynamics within a basaltic conduit revealed by textural and compositional features of erupted ash: The December 2015 Mt. Etna paroxysms. *Sci. Rep.* **7**, 4805 (2017).

20. L. Costantini, B. F. Houghton, C. Bonadonna, Constraints on eruption dynamics of basaltic explosive activity derived from chemical and microtextural study: The example of the Fontana Lapilli Plinian eruption, Nicaragua. *J. Volcanol. Geotherm. Res.* **189**, 207–224 (2010).

21. F. Alfano, M. H. Ort, L. Pioli, S. Self, S. L. Hanson, K. Roggensack, C. M. Allison, R. Amos, A. B. Clarke, Subplinian monogenetic basaltic eruption of Sunset Crater, Arizona, USA. *Bull. Geol. Soc. Am.* **131**, 661–674 (2019).

22. J. T. Caulfield, S. J. Cronin, S. P. Turner, L. B. Cooper, Mafic Plinian volcanism and ignimbrite

emplacement at Tofua volcano, Tonga. *Bull. Volcanol.* **73**, 1259–1277 (2011).

23. S. Campagnola, C. Romano, L. G. Mastin, A. Vona, *Confort 15* model of conduit dynamics : Applications to Pantelleria Green Tuff and Etna 122 BC eruptions. *Contrib. to Mineral. Petrol.* **171**, 60 (2016).

24. C. Romano, A. Vona, S. Campagnola, G. Giordano, I. Arienzo, R. Isaia, Modelling and physico-chemical constraints to the 4.5 ka Agnano-Monte Spina Plinian eruption (Campi Flegrei, Italy). *Chem. Geol.* **532**, 119301 (2020).

25. H. Sigurdsson, S. Carey, Plinian and co-ignimbrite tephra fall from the 1815 eruption of Tambora volcano. *Bull. Volcanol.* **51**, 243–270 (1989).

26. H. M. Mader, E. W. Llewellin, S. P. Mueller, The rheology of two-phase magmas: A review and analysis. *J. Volcanol. Geotherm. Res.* **257**, 135–158 (2013).

27. D. Ayalew, P. Barbey, B. Marty, L. Reisberg, G. Yirgu, R. Pik, Source, genesis, and timing of giant ignimbrite deposits associated with Ethiopian continental flood basalts. *Geochim. Cosmochim. Acta* **66**, 1429–1448 (2002).

28. K. D. Burgess, R. M. Stroud, M. Darby Dyar, M. C. Mccanta, Submicrometer-scale spatial heterogeneity in silicate glasses using aberration-corrected scanning transmission electron microscopy. *Am. Mineral.* **101**, 2677–2688 (2016).

29. G. Barone, P. Mazzoleni, R. A. Corsaro, P. Costagliola, F. Di Benedetto, E. Ciliberto, D. Gimeno, C. Bongiorno, C. Spinella, Nanoscale surface modification of Mt. Etna volcanic ashes. *Geochim. Cosmochim. Acta* **174**, 70–84 (2016).

30. D. Di Genova, A. Caracciolo, S. Kolzenburg, Measuring the degree of "nanotilization" of volcanic glasses: Understanding *syn*-eruptive processes recorded in melt inclusions. *Lithos* **318–319**, 209–218 (2018).

31. M. Mujin, M. Nakamura, A. Miyake, Eruption style and crystal size distributions: Crystallization of groundmass nanolites in the 2011 Shinmoedake eruption. *Am. Mineral.* **102**, 2367–2380 (2017).

32. C. Liebske, H. Behrens, F. Holtz, R. A. Lange, The influence of pressure and composition on the viscosity of andesitic melts. *Geochim. Cosmochim. Acta* **67**, 473–485 (2003).

33. D. Di Genova, S. Sicola, C. Romano, A. Vona, S. Fanara, L. Spina, Effect of iron and nanolites on Raman spectra of volcanic glasses: Reassessment of existing strategies to estimate the water content. *Chem. Geol.* **475**, 76–86 (2017).

34. P. W. Voorhees, The theory of Ostwald ripening. *J. Stat. Phys.* **38**, 231–252 (1985).

35. V. Y. Rudyak, Viscosity of nanofluids. Why it is not described by the classical theories. *Adv. Nanoparticles.* **2**, 266–279 (2013).

36. R. Taylor, S. Coulombe, T. Otanicar, P. Phelan, A. Gunawan, W. Lv, G. Rosengarten, R. Prasher, H. Tyagi, Small particles, big impacts: A review of the diverse applications of nanofluids. *J. Appl. Phys.* **113**, 011301 (2013).

37. P. Pleše, M. D. Higgins, D. R. Baker, G. Lanzafame, M. K. Prašek, L. Mancini, S. M. Rooyakkers, Production and detachment of oxide crystal shells on bubble walls during experimental vesiculation of andesitic magmas. *Contrib. Mineral. Petrol.* **174**, 21 (2019).

38. D. Di Genova, A. Zandona, J. Deubener, Unravelling the effect of nano-heterogeneity on the viscosity of silicate melts: Implications for glass manufacturing and volcanic eruptions. *J. Non Cryst. Solids* **545**, 120248 (2020).

39. D. Di Genova, S. Kolzenburg, S. Wiesmaier, E. Dallanave, D. R. Neuville, K.-U. Hess, D. B. Dingwell, A chemical tipping point governing mobilization and eruption style of rhyolitic magma. *Nature* **552**, 235–238 (2017).

40. J. L. Knipping, J. D. Webster, A. C. Simon, F. Holtz, Accumulation of magnetite by flotation on bubbles during decompression of silicate magma. *Sci. Rep.* **9**, 3852 (2019).

41. F. Arzilli, G. La Spina, M. R. Burton, M. Polacci, N. Le Gall, M. E. Hartley, D. Di Genova, B. Cai, N. T. Vo, E. C. Bamber, S. Nonni, R. Atwood, E. W. Llewellin, R. A. Brooker, H. Mader, P. D. Lee, Magma fragmentation in highly explosive basaltic eruptions induced by rapid crystallization. *Nat.*

Geosci. **12**, 1023–1028 (2019).

42. B. Giaccio, I. Arienzo, G. Sottili, F. Castorina, M. Gaeta, S. Nomade, P. Galli, P. Messina, Isotopic (Sr-Nd) and major element fingerprinting of distal tephras: An application to the Middle-Late Pleistocene markers from the Colli Albani volcano, central Italy. *Quat. Sci. Rev.* **67**, 190–206 (2013).

43. A. Di Muro, D. Giordano, B. Villemant, G. Montagnac, B. Scaillet, C. Romano, Influence of composition and thermal history of volcanic glasses on water content as determined by micro-Raman spectrometry. *Appl. Geochemistry.* **21**, 802–812 (2006).

44. G. A. R. Gualda, M. S. Ghiorso, R. V. Lemons, T. L. Carley, Rhyolite-MELTS: A modified calibration of MELTS optimized for silica-rich, fluid-bearing magmatic systems. *J. Petrol.* **53**, 875–890 (2012).

45. L.-C. Kuo, R. J. Kirkpatrick, Small angle X-ray scattering study of pre-nucleation behavior of titanium-free and titanium-bearing diopside glasses. *Am. Mineral.* **67**, 676–685 (1982).

46. K. Goepfert, J. E. Gardner, Influence of pre-eruptive storage conditions and volatile contents on explosive Plinian style eruptions of basic magma. *Bull. Volcanol.* **72**, 511–521 (2010).

47. D. Di Genova, C. Romano, D. Giordano, M. Alletti, Heat capacity, configurational heat capacity and fragility of hydrous magmas. *Geochim. Cosmochim. Acta* **142**, 314–333 (2014).

48. T. M. Stawski, D. B. van den Heuvel, R. Besselink, D. J. Tobler, L. G. Benning, Mechanism of silica-lysozyme composite formation unravelled by in situ fast SAXS. *Beilstein J. Nanotechnol.* **10**, 182–197 (2019).

49. D. Giordano, J. K. Russell, D. B. Dingwell, Viscosity of magmatic liquids: A model. *Earth Planet. Sci. Lett.* **271**, 123–134 (2008).

50. F. Cáceres, F. B. Wadsworth, B. Scheu, M. Colombier, C. Madonna, C. Cimarelli, K.-U. Hess, M. Kaliwoda, B. Ruthensteiner, D. B. Dingwell, Can nanolites enhance eruption explosivity? *Geology* **48**, 10.1130/G47317.1 (2020).

51. A. Orlando, M. D. Orazio, P. Armienti, D. Borrini, C. N. R. Istituto, U. O. Firenze, G. La Pira, U. Pisa, S. Maria, M. D'Orazio, P. Armienti, D. Borrini, Experimental determination of plagioclase and clinopyroxene crystal growth rates in an anhydrous trachybasalt from Mt Etna (Italy). *Eur. J. Mineral.* **20**, 653–664 (2008).

52. H. Cabane, D. Laporte, A. Provost, An experimental study of Ostwald ripening of olivine and plagioclase in silicate melts: Implications for the growth and size of crystals in magmas. *Contrib. Mineral. Petrol.* **150**, 37–53 (2005).

53. P. Del Gaudio, S. Mollo, G. Ventura, G. Iezzi, J. Taddeucci, A. Cavallo, Cooling rate-induced differentiation in anhydrous and hydrous basalts at 500 MPa: Implications for the storage and transport of magmas in dikes. *Chem. Geol.* **270**, 164–178 (2010).

54. A. S. Lloyd, P. Ruprecht, E. H. Hauri, W. I. Rose, H. M. Gonnermann, T. Plank, NanoSIMS results from olivine-hosted melt embayments: Magma ascent rate during explosive basaltic eruptions. *J. Volcanol. Geotherm. Res.* **283**, 1–18 (2014).

55. L. A. Szramek, Mafic Plinian eruptions: Is fast ascent required? *J. Geophys. Res. Solid Earth* **121**, 7119–7136 (2016).

56. M. Cassidy, M. Manga, K. V. Cashman, O. Bachmann, Controls on explosive-effusive volcanic eruption styles. *Nat. Commun.* **9**, 2839 (2018).

57. J. Chevalier, O. Tillement, F. Ayela, Rheological properties of nanofluids flowing through microchannels. *Appl. Phys. Lett.* **91**, 233103 (2007).

58. K. Yapici, O. Osturk, Y. Uludag, Dependency of nanolfuid rheology on particle size and concentration of various metal oxide nanoparticles. *Braz. J. Chem. Eng.* **35**, 575–586 (2018).

59. S. M. S. Murshed, P. Estellé, A state of the art review on viscosity of nanofluids. *Renew. Sustain. Energy Rev.* **76**, 1134–1152 (2017).

60. V. S. Raghuwanshi, R. Harizanova, D. Tatchev, A. Hoell, C. Rüssel, Structural analysis of Fe-Mn-O nanoparticles in glass ceramics by small angle scattering. *J. Solid State Chem.* **222**, 103–110 (2015).

61. L. Bergström, Shear thinning and shear thickening of concentrated ceramic suspensions. *Colloids Surfaces A Physicochem. Eng. Asp.* **133**, 151–155 (1998).

62. D. J. M. Burkhard, T. Schaller, The effect of initial oxidation state on crystallization of basaltic glass. *J. Non Cryst. Solids* **352**, 3961–3969 (2006).

63. A. A. Morrison, M. Zanetti, C. W. Hamilton, E. Lev, C. D. Neish, A. G. Whittington, Rheological investigation of lunar highland and mare impact melt simulants. *Icarus* **317**, 307–323 (2019).

64. P. Del Gaudio, G. Ventura, J. Taddeucci, The effect of particle size on the rheology of liquid-solid mixtures with application to lava flows: Results from analogue experiments. *Geochemistry. Geophys. Geosystems.* **14**, 2661–2669 (2013).

65. T. M. Stawski, A. E. S. Van Driessche, M. Ossorio, J. Diego Rodriguez-Blanco, R. Besselink, L. G. Benning, Formation of calcium sulfate through the aggregation of sub-3 nanometre primary species. *Nat. Commun.* **7**, 11177 (2016).

66. E. C. Sklute, S. Kashyap, M. D. Dyar, J. F. Holden, T. Tague, P. Wang, S. J. Jaret, Spectral and morphological characteristics of synthetic nanophase iron (oxyhydr)oxides. *Phys. Chem. Miner.* **45**, 1–26 (2018).

67. G. N. Greaves, EXAFS and the structure of glass. *J. Non Cryst. Solids* **71**, 203–217 (1985).

68. J. W. P. Schmelzer, Crystal nucleation and growth in glass-forming melts: Experiment and theory. *J. Non Cryst. Solids* **354**, 269–278 (2008).

69. D. R. Neuville, L. Hennet, P. Florian, D. de Ligny, In situ high-temperature experiments. *Rev. Mineral. Geochem.* **78**, 779–800 (2014).

70. M. Polacci, F. Arzilli, G. La Spina, N. Le Gall, B. Cai, M. E. Hartley, D. Di Genova, N. T. Vo, S. Nonni, R. C. Atwood, E. W. Llewellin, P. D. Lee, M. R. Burton, Crystallisation in basaltic magmas revealed via *in situ* 4D synchrotron X-ray microtomography. *Sci. Rep.* **8**, 8377 (2018).

71. A. C. Larson, R. B. Von Dreele, General Structure Analysis System (GSAS), LANL Report LAUR

86-748 (2004).

72. J. W. E. Drewitt, C. Sanloup, A. Bytchkov, S. Brassamin, L. Hennet, Structure of $(Fe_xCa_{1-x}O)_y(SiO_2)_{1-y}$ liquids and glasses from high-energy X-ray diffraction: Implications for the structure of natural basaltic magmas. *Phys. Rev. B.* **87**, 224201 (2013).

73. M. Basham, J. Filik, M. T. Wharmby, P. C. Y. Chang, B. E. Kassaby, M. Gerring, J. Aishima, K. Levik, B. C. A. Pulford, I. Sikharulidze, D. Sneddon, M. Webber, S. S. Dhesi, F. Maccherozzi, O. Svensson, S. Brockhauser, G. Náray, A. W. Ashton, Data analysis WorkbeNch (DAWN). *J. Synchrotron. Rad.* **22**, 853–858 (2015).

74. M. Doucet, J. H. Cho, G. Alina, J. Bakker, W. Bouwman, P. Butler, K. Campbell, M. Gonzales, R. Heenan, A. Jackson, P. Juhas, S. King, P. Kienzle, J. Krzywon, A. Markvardsen, T. Nielsen, L. O'Driscoll, W. Potrzebowski, R. Ferraz Leal, T. Richter, P. Rozycko, A. Washington, SasView version 4.1 (2017).

75. I. M. Krieger, T. J. Dougherty, A mechanism for Non-Newtonian flow in suspensions of rigid spheres. *J. Rheol.* **3**, 137–152 (1959).

76. M. Schanofski, S. Fanara, B. C. Schmidt, CO_2–H_2O solubility in K-rich phonolitic and leucititic melts. *Contrib. Mineral. Petrol.* **174**, 1–18 (2019).

77. A. P. Zav'yalov, V. V. Syzrantsev, K. V. Zobov, S. P. Bardakhanov, Influence of agglomeration on the viscosity of nanofluids. *J. Eng. Phys. Thermophys.* **91**, 115–123 (2018).

78. E. E. Michaelides, *Nanofluidics: Thermodynamic and Transport Properties* (Springer International Publishing, 2014).

79. D. Di Genova, J. Vasseur, K.-U. Hess, D. R. Neuville, D. B. Dingwell, Effect of oxygen fugacity on the glass transition, viscosity and structure of silica- and iron-rich magmatic melts. *J. Non Cryst. Solids* **470**, 78–85 (2017).
